# ErbB2/Her2-dependent downregulation of a cell death-promoting protein BLNK in breast cancer cells is required for 3D breast tumor growth

**DOI:** 10.1038/s41419-022-05117-9

**Published:** 2022-08-06

**Authors:** Xiaoyang Liu, Sandhya Chipurupalli, Peijia Jiang, Mahtab Tavasoli, Byong Hoon Yoo, Michael McPhee, Sina Mazinani, Giulio Francia, Robert S. Kerbel, Kirill V. Rosen

**Affiliations:** 1 Departments of Pediatrics & Biochemistry and Molecular Biology, Dalhousie University, Halifax, NS Canada; 2 Department of Pharmacology, Department of Pediatrics, Dalhousie University, Halifax, NS Canada; 3 Border Biomedical Research Center, University of Texas at El Paso (UTEP), El Paso, TX USA; 4 Biological Sciences Platform, Sunnybrook Research Institute, Toronto, ON Canada; 5 University of Toronto Department of Medical Biophysics, Toronto, ON Canada

**Keywords:** Cancer, Cell biology

## Abstract

A significant proportion of breast cancers are driven by ErbB2/Her2 oncoprotein that they overexpress. These malignancies are typically treated with various ErbB2-targeted drugs, but many such cancers develop resistance to these agents and become incurable. Conceivably, treatment of ErbB2-positive cancers could be facilitated by use of agents blocking oncogenic signaling mechanisms downstream of ErbB2. However, current understanding of these mechanisms is limited. The ability of solid tumor cells to resist anoikis, cell death triggered by cell detachment from the extracellular matrix (ECM), is thought to be critical for 3D tumor growth. In an effort to understand the mechanisms of ErbB2-driven breast cancer cell anoikis resistance we found that detachment of non-malignant breast epithelial cells from the ECM upregulates a cell death-promoting tumor suppressor adapter protein BLNK and that ErbB2 blocks this upregulation by reducing tumor cell levels of transcription factor IRF6. We further observed that trastuzumab, a therapeutic anti-ErbB2 antibody, upregulates BLNK in human trastuzumab-sensitive but not trastuzumab-resistant ErbB2-positive breast cancer cells. Moreover, we established that BLNK promotes anoikis by activating p38 MAP kinase and that ErbB2-dependent BLNK downregulation blocks breast cancer cell anoikis. In search for pharmacological approaches allowing to upregulate BLNK in tumor cells we found that clinically approved proteasome inhibitor bortezomib upregulates IRF6 and BLNK in human breast cancer cells and inhibits their 3D growth in a BLNK-dependent manner. In addition, we found that BLNK upregulation in human ErbB2-positive breast cancer cells blocks their ability to form tumors in mice. Furthermore, we used publicly available data on mRNA levels in multiple breast cancers to demonstrate that increased BLNK mRNA levels correlate with increased relapse-free survival in a cohort of approximately 400 patients with ErbB2-positive breast cancer. In summary, we discovered a novel mechanism of ErbB2-driven 3D breast tumor growth mediated by ErbB2-dependent BLNK downregulation.

## Introduction

Approximately 15% of breast cancers overexpress ErbB2/Her2 receptor tyrosine kinase [1] and are normally treated with ErbB2-targeted drugs [2, 3]. Approximately 20% of such malignancies develop resistance to these agents and become incurable [2, 3]. Conceivably, treatment efficacy of ErbB2-positive cancers could be facilitated by drugs blocking oncogenic mechanisms downstream of ErbB2. However, current understanding of these mechanisms is limited.

One critical feature of breast tumors is their ability to grow as 3D masses [4]. To grow in this manner, cancer cells need to survive without adhesion to the extracellular matrix (ECM) [4]. This is due to the fact that normal breast epithelial cells are attached to the ECM in the mammary gland, and detachment kills them [4]. This type of death is called anoikis [5]. In contrast, breast tumors grow and metastasize as three-dimensional masses in which the cells remain viable without being properly attached to the ECM [6]. Numerous studies indicate that cancer cell anoikis resistance is critical for tumor progression. For example, tumor cell ability to survive and grow without attachment to the ECM as colonies in soft agar is a ″gold standard″ for oncogenic transformation [7]. In addition, major oncoproteins, including ErbB2 [8] and EGFR [9], inhibit anoikis. Moreover, approaches triggering anoikis of cancer cells inhibit their tumorigenicity and metastatic capacity [10].

ErbB2 inhibits breast cancer cell anoikis by partially understood mechanisms. We discovered recently that one such major mechanism is driven by ErbB2-dependent downregulation of transcription factor IRF6 [11], a member of the Interferon Regulatory Factor transcription factor family [12]. IRF6 is upregulated in the breast during mammary gland involution [13], and such involution is likely mediated by breast epithelial cell anoikis [14]. We found that ErbB2 downregulates IRF6 in breast cancer cells by activating a protein kinase MEK and its target protein kinase ERK [11].

The mechanisms by which IRF6 controls anoikis are unknown. We show here that ErbB2-dependent IRF6 downregulation reduces cellular levels of the pro-apoptotic protein BLNK [15] and that BLNK loss blocks anoikis of breast cancer cells and promotes their tumorigenicity in vivo.

## Materials and methods

### Materials

The following compounds were used. SB203580 (Sigma–Aldrich, St. Louis, MO, USA), trastuzumab (Roche, Mississauga, ON, Canada), bortezomib Santa Cruz Biotechnology (Santa Cruz, CA, USA) Matrigel (VWR, Mississauga, ON, Canada), HBSS medium (Sigma–Aldrich).

### Expression vectors

BLNK cDNA in pcDNA3.1 was from Genscript (Piscataway, NJ, USA). pBABE-hygro vector was from Addgene (Cambridge, MA, USA). pRetroX-TetOne™-Puro expression vector was from Takara Bio (Mountain View, CA, USA). To generate pRetroX-TetOne™-Puro-BLNK expression vector the BLNK-encoding sequence was excised from pcDNA3.1 by EcoRI and BamHI restriction enzymes and inserted using T4 DNA ligase in EcoRI- and BamHI-digested pRetroX-TetOne™-Puro. To generate BLNK pRetroX-TetOne™-Hygro-BLNK expression vector, pBabeHygro vector was digested with MluI and SfiI restriction enzymes, the hygromycin resistance gene was blunt-ended with the Klenow DNA polymerase fragment and inserted by use of T4 DNA ligase in BLNK-encoding pRetroX-TetOne™-Puro expression vector from which the puromycin resistance gene was excised using SfiI and EcoRV restriction enzymes. To generate pBABE-hygro-BLNK expression vector, BLNK-encoding sequence was excised from pcDNA3.1, blunt-ended as described above and inserted by use of T4 DNA ligase in BsaAI restriction enzyme-digested pBABE-hygro vector. Generation of pBabe-hygro-IRF6 expression vector was published [11]. pHIT and pVSVG retroviral vectors were provided by P. Lee (Dalhousie University). pBABE-hygro expression vector was from Addgene. pSPAX2 and pMD2.G were provided by L. Attardi (Stanford University).

### Cell culture

MCF-10A cells and their derivatives MCF-ErbB2 and MCF-MekDD cells were provided by M. Reginato (Drexel University, USA) [16]. MCF10A cells were authenticated as published [11]. Lack of mycoplasma contamination in all cells was established as published [11]. BT-474 (American Type Culture Collection (ATCC), Manassas, VA, USA), BT474TR and BT474T cells were cultured as published [11]. Generation of BT-474TR cells is published [17]. To generate tumorigenic BT474T cells, BT474 cells were implanted orthotopically into the mammary fat pad of severe combined immunodeficiency mouse, and resulting tumors were serially passaged into new hosts over a three-year period [18]. AU-565 (ATCC), SKBR3 (ATCC) and 293 T cells (provided by A. Stadnyk, Dalhousie University) were cultured as published [11]. To detach the cells from the ECM, they were plated in suspension culture above a layer of 1% sea plaque agarose polymerized in respective culture medium not containing any additional ingredients.

### Antibodies

Anti-IRF6 (cat# 6948 S), anti-BLNK (cat# 36438), anti-p38MAPK (cat# 9212 S), anti-phospho-p38MAPK (cat# 4511 T), anti-caspase-3 (cat# 9962 S), anti-JNK (cat# 9255 S), anti-phospho-JNK (cat# 9252 S), anti-Jak3 (cat# 5481) and anti-α-tubulin (cat# 3873) were from Cell Signalling Technology, Danvers, MA, USA.

### RNA interference

Small interfering (si)RNAs (Horizon Discovery, Lafayette, CO, USA) were used as described [19]. BLNK small hairpin (sh)RNA-encoding lentiviral vectors were from Sigma–Aldrich (St. Louis, MO, USA). To generate BLNK-deficient cells, 3 × 10^6^ GP2-293 cells were incubated with 3 ug pLKO control shRNA or pLKO-Blnk shRNA 7, or 8 vectors in the presence of 2.25 ug psPAX2 and 0.75 ug pMD2.G vectors and 50 μl of Lipofectamine 2000 in 6 ml of OPTI-MEM. The medium was changed 4 h later to DMEM containing 10% FBS. The medium was collected 48 h later and filtered through a 0.45-micron filter. 3 ml of the resulting solution were added to 10^6^ BT474 cells together with 8 μg/ml polybrene. Medium was changed 24 h later to the fresh medium, the cells were cultured for 72 h in the presence of 2 μg/ml puromycin and expanded as stable cell lines.

### Transduction of cells with retroviruses

To generate BLNK-overproducing MCF-ErbB2 cells, 3 × 10^6^ 293 T cells were incubated with 14 μg of control pBabehygro expression vector or pBabehygro-BLNK vector and 7 μg of pHIT and 7 μg of pVSVG vectors encoding retroviral proteins in the presence of 50 μl of Lipofectamine 2000 (Invitrogen, Carlsbad, CA, USA) in 6 ml of Opti-MEM. BLNK-overproducing cells were generated as published [11]. Irf6-overproducing MCF-ErbB2 cells were generated as described above but pBabe-IRF6 expression vector was used. To generate MCF-ErbB2 cells producing doxycycline-inducible BLNK, pRetroX-TetOne™-Hygro and pRetroX-TetOne™-Hygro-BLNK vectors were used as described above. To generate BT474T cells producing doxycycline-inducible BLNK, pRetroX-TetOne™-PURO and pRetroX-TetOne™-PURO- BLNK vectors were used as described above.

### Flow cytometry

Cells were analyzed by use of PE Annexin V Apoptosis Detection Kit I, BD Pharmingen (San Diego, CA, USA) according to manufacturer’s instructions.

### Orthotopic tumor implantation

Animal studies were approved by Dalhousie University Committee on Laboratory Animals. Female 6-week-old Nu/Nu Nude mice (Charles River Canada, Saint-Constant, QC) were allowed to acclimatize for 2 weeks. 8 × 10^6^ cells were harvested by trypsin treatment, washed thrice in ice-cold PBS, resuspended in a 100 μL of 1:1 mixture of PBS and Matrigel and injected into the inguinal mammary fat pad. 2 mg/ml doxycycline was added or not to the animals’ drinking water. Tumor volumes were measured as published [20].

### Statistical analysis

Statistical analysis of the data in Supplementary fig. 4C, 6C and 8 was performed by the two-sided chi-square test for goodness-of-fit and statistical analysis of all other data, by the two-sided Student’s t-test.

Western blotting [21], qPCR and detection of clonogenic cell survival were performed as published [11]. Western blot quantification was performed by Odyssey or Image J software and is shown shown as supplementary data along with the original blots.

Sequences of siRNAs, shRNAs and DNA primers used in the study are shown in Supplementary Table 1.

## Results

### ErbB2 downregulates BLNK in breast epithelial cells

We found previously that ErbB2 blocks breast cancer cell anoikis by downregulating transcription factor IRF6 [11]. Others applied gene expression microarray analysis to identify mRNAs whose expression is altered by IRF6 knockdown in human keratinocytes by RNA interference (RNAi) [22]. One mRNA downregulated by IRF6 knockdown was that encoding the cell death-promoting adapter protein BLNK. BLNK carries multiple tyrosine phosphorylation sites, a C-terminal SH2 domain and a central proline-rich region that binds SH3 domains of other proteins [15]. BLNK has mainly been studied in the context of B cell signalling [15]. Upon activation by B cell receptor, a tyrosine protein kinase Syk phosphorylates BLNK on various tyrosine residues serving as binding sites for enzymes PLCγ, Vav and BTK as well as those for linker proteins, e.g., Grb2 and Nck. BLNK-bound proteins can in turn activate pro-apoptotic protein kinases JNK and p38MAP kinase (p38MAPK) [15, 23]. It was found that BLNK-deficient mice spontaneously develop pre-B-cell leukemia [23].

**Fig. 1:**
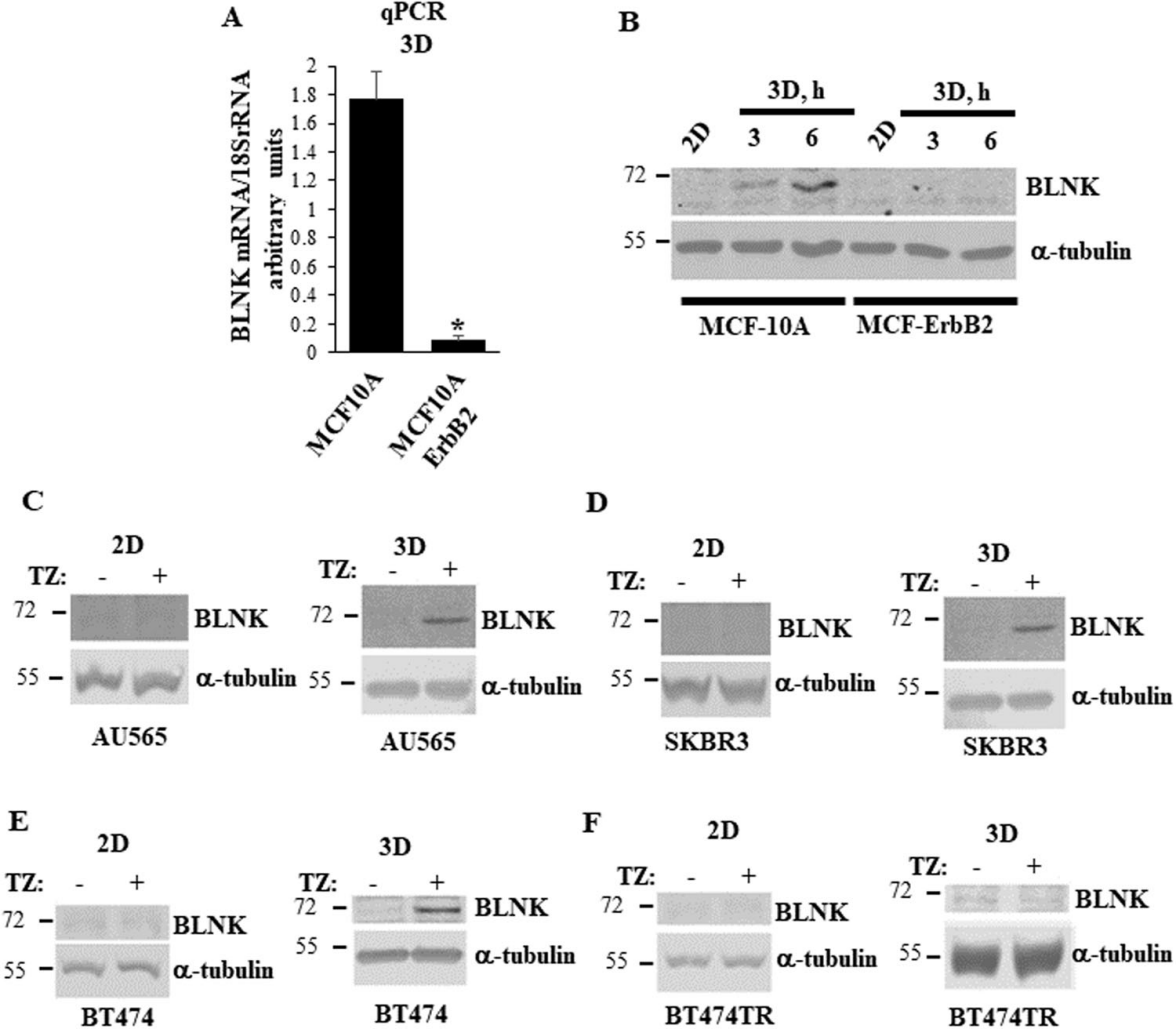
ErbB2 downregulates BLNK in detached breast cancer cells. **A** MCF10A and MCF-ErbB2 cells were cultured detached from the ECM (3D) for 24 h and BLNK mRNA levels were analyzed in the cells by quantitative PCR (qPCR). BLNK mRNA levels normalized by those of 18 S rRNA (determined by qPCR) expressed in arbitrary units are shown. Results represent the average of three independent experiments plus the SD. *p-value is < 0.05. **B** MCF10A and MCF-ErbB2 cells were cultured attached to (2D) or detached from (3D) the ECM for the indicated times and assayed for BLNK levels by western blotting. α-tubulin was used as a loading control. **C**–**E** Human ErbB2-positive cell lines AU565, SKBR3 and BT474 were cultured attached to (2D) (left) or detached from the ECM (3D) (right) for 48 h in the absence (−) or in the presence (+) of 5 μg/ml trastuzumab (TZ) and assayed as in (**B**). **F** Trastuzumab-resistant variant of BT474 cells BT474TR was assayed as in (**C**–**E**).

In an effort to identify the mechanisms by which IRF6 controls breast cancer cell anoikis we used spontaneously immortalized anoikis-susceptible human non-malignant breast epithelial cells MCF10A and their anoikis-resistant derivative MCF-ErbB2 generated by infection of MCF10A cells with the wild type ErbB2-encoding retrovirus [16]. We found that ErbB2 strongly downregulates BLNK mRNA in detached MCF10A cells (Fig. 1A). Moreover, detachment of MCF-10A cells upregulated BLNK protein, and ErbB2 blocked this upregulation (Fig. 1B).

The effect of ErbB2 on BLNK was not unique to MCF10A cells since we found that while treatment of attached human ErbB2-positive breast cancer cells AU565, SKBR3 and BT474 [11] with the therapeutic anti-ErbB2 antibody trastuzumab does not upregulate BLNK protein (Fig. 1C–E, left), trastuzumab noticeably upregulates this protein when these cells are detached from the ECM (Fig. 1C–E, right). In contrast, trastuzumab failed to upregulate BLNK in a trastuzumab-resistant variant of BT474 cells BT474TR [17] regardless of whether these cells were attached to or detached from the ECM (Fig. 1F). Thus, ErbB2 downregulates BLNK in detached breast cancer cells.

### Exogenous IRF6 upregulates BLNK in detached breast cancer cells

**Fig. 2:**
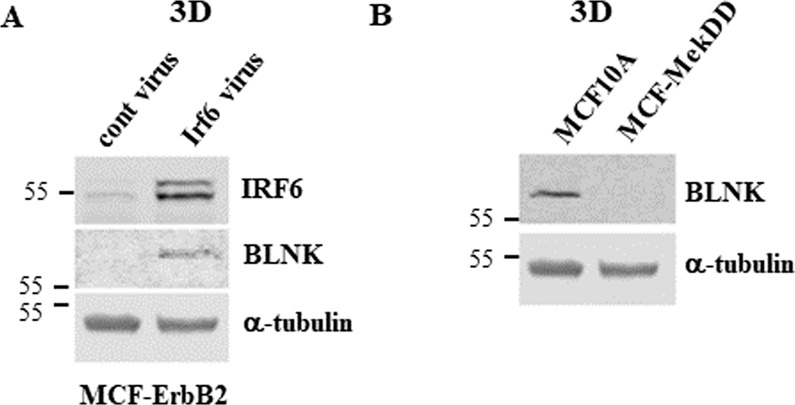
Irf6 upregulates BLNK in detached ErbB2-positive breast cancer cells. **A** MCF-ErbB2 cells infected with the control (cont virus) or the Irf6-encoding Maloney murine leukemia virus (MMLV) (Irf6 virus) were cultured detached from the ECM for 24 h and assayed for Irf6 and BLNK levels by western blotting. α-tubulin was used as a loading control. **B** MCF10A cells and a variant of MCF10A cells obtained by infection of these cells with a retrovirus carrying constitutively active Mek2 mutant (MCF-MekDD) were cultured detached from the ECM for 24 h and assayed for BLNK expression as in **A**.

To test whether ErbB2-induced BLNK downregulation is mediated by IRF6 downregulation we infected MCF-ErbB2 cells, in which IRF6 is downregulated by ErbB2, with the IRF6-encoded retrovirus (Fig. 2A). We observed that IRF6 upregulates BLNK in these cells (Fig. 2A). Thus, ErbB2 reduces BLNK levels in the indicated cells by downregulating IRF6. Noteworthily, while others found that IRF6 knockdown by RNAi in human keratinocytes downregulates BLNK, the same study used chromatin immunoprecipitation-sequencing analysis to demonstrate that IRF6 does not bind genomic DNA adjacent to the BLNK gene [22]. Hence, IRF6 does not directly control the BLNK gene transcription but likely regulates transcription of gene(s) that affect cellular BLNK levels.

We found previously that ErbB2 downregulates IRF6 in breast cancer cells by activating protein kinase MEK [11]. In agreement with these data, we observed now that expression of an activated MEK mutant in MCF10A cells mimics the effect of ErbB2 on BLNK (Fig. 2A). In summary, ErbB2-induced BLNK downregulation in detached breast cancer cells is driven by IRF6 downregulation.

### BLNK contributes to anoikis of non-malignant breast epithelial cells

**Fig. 3:**
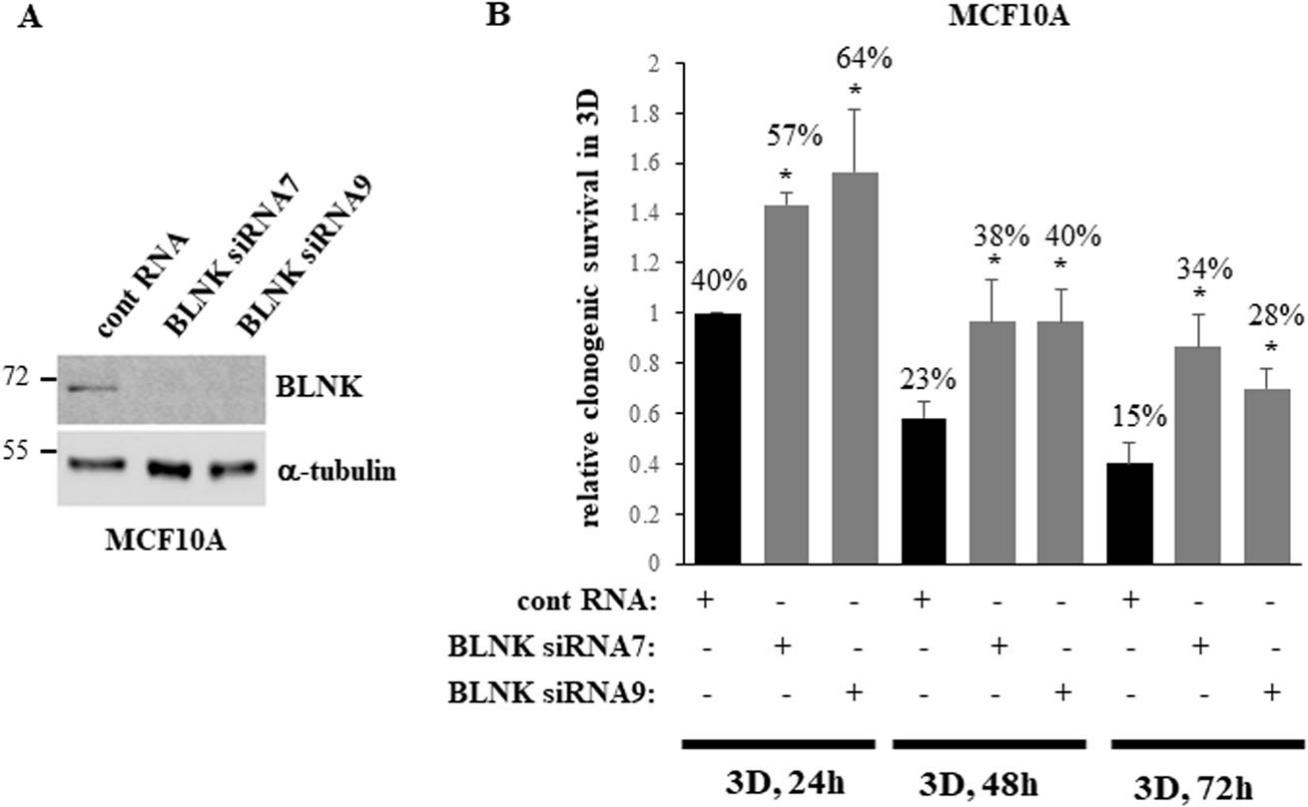
Detachment-induced BLNK upregulation is required for anoikis of non-malignant breast epithelial cells. **A** MCF10A cells transfected with 100 nM control RNA (cont RNA) or BLNK-specific siRNA (BLNK siRNA) 7 or 9 were detached for 3 h (3D) and assayed for BLNK expression by western blotting. α-tubulin was used as a loading control. **B** MCF10A cells treated as in **A** were allowed to form colonies in monolayer immediately or after being detached for the indicated times. The ratio of colony number formed by the cells detached for each time period to that formed by the cells plated in monolayer immediately after transfection is shown in each case. This ratio observed for the cells detached for 24 h was designated as 1.0. The data represent the average of three independent experiments plus the SD.

We observed that detachment of non-malignant anoikis-sensitive breast epithelial cells MCF10A from the ECM upregulates BLNK (Fig. 1B). To establish whether BLNK mediates anoikis of MCF10A cells, we knocked BLNK down by two different BLNK-specific siRNAs in these cells (Fig. 3A). We noticed that while detachment from the ECM triggered a strong loss of clonogenic survival of the control MCF10A cells, BLNK knockdown substantially delayed this loss (Fig. 3B). Thus, detachment-induced BLNK upregulation contributes to anoikis of non-malignant breast epithelial cells.

### ErbB2-induced BLNK downregulation is required for anoikis resistance of breast epithelial cells

**Fig. 4:**
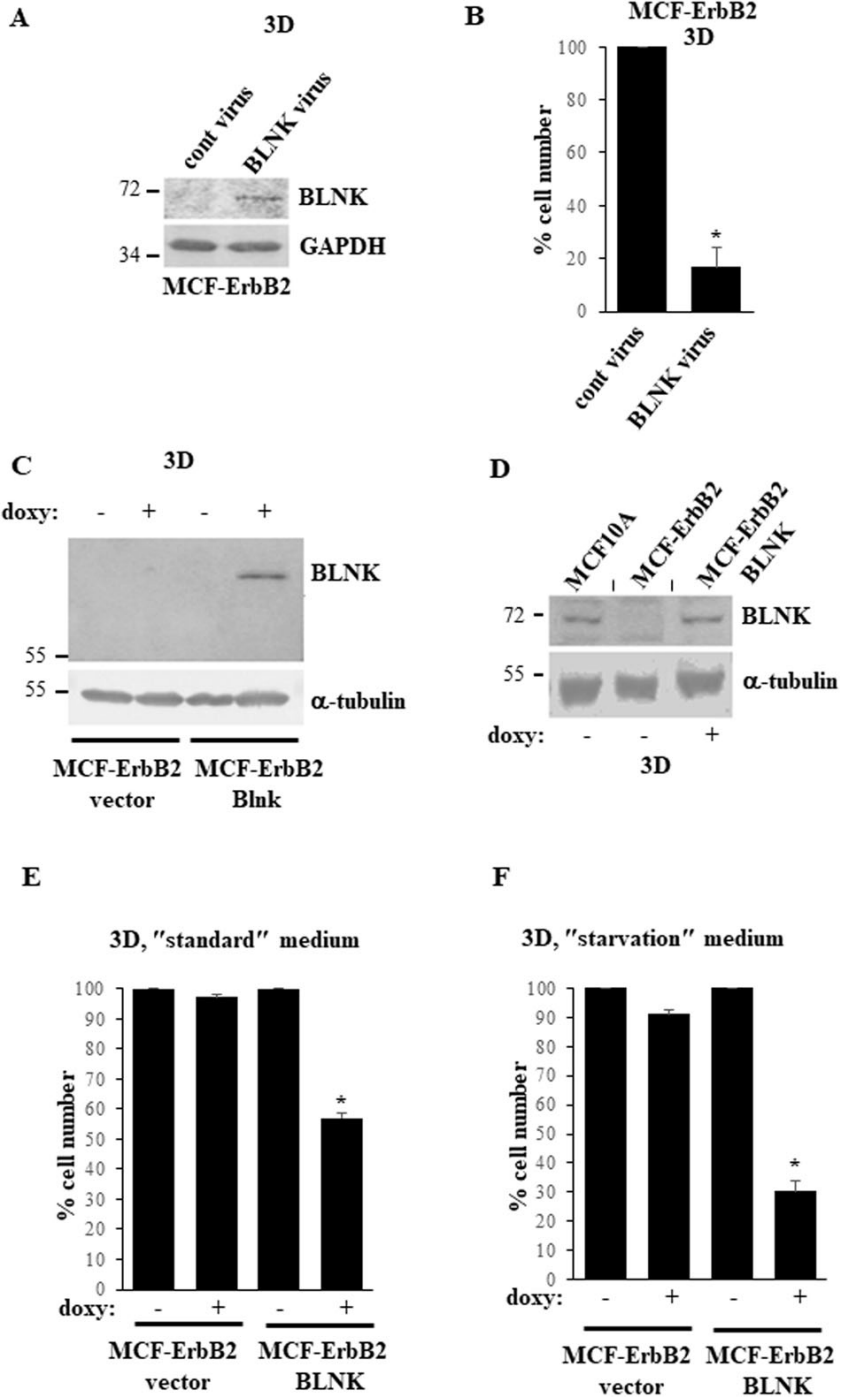
Downregulation of BLNK is required for ErbB2-induced 3D growth of breast cancer cells. **A** MCF-ErbB2 cells infected with the control (cont virus) or the BLNK-encoding MMLV (BLNK virus) were cultured detached from the ECM for 24 h (3D) and assayed for BLNK levels by western blotting. GAPDH was used as a loading control. **B** MCF-ErbB2 cells treated as in **A** were detached from the ECM for 72 h (3D) and counted. The number of cells detected in the control sample was designated as 100%. **C** Indicated cell lines were cultured detached from the ECM (3D) for 48 h in the absence (−) or in the presence (+) of 5 ng/ml doxycycline (doxy) and assayed for BLNK expression by western blotting. α-tubulin was used as a loading control. **D** Indicated cell lines were cultured detached from the ECM (3D) for 6 h in the absence (−) or in the presence (+) of 5 ng/ml doxycycline (doxy) and assayed as in **C**. A short vertical black line was used to indicate that lanes were removed from the image and separate parts of an image were joined together. **E** Indicated cell lines were cultured for 72 h as in **C** in the ″standard″ medium (Hybri-Care medium supplemented with 10% Fetal Bovine Serum) used for culturing these cells in all experiments except for **F** and counted. The number of cells detected in the control sample was designated as 100%. **F** Cells cultured in the ″starvation″ medium which represented the Hybri-Care medium medium supplemented with 0.1% Fetal Bovine Serum and diluted 2.5-fold with HBSS medium were assayed as in **E**. The data in **B**, **E**, **F** are the average of the three independent experiments plus the SD. *p-value < 0.05.

To reverse the effect of ErbB2 on BLNK we infected MCF-ErbB2 cells with a BLNK-encoding retrovirus (Fig. 4A). We observed that exogenous BLNK noticeably reduced the number of detached MCF-ErbB2 cells (Fig. 4B).

**Fig. 5:**
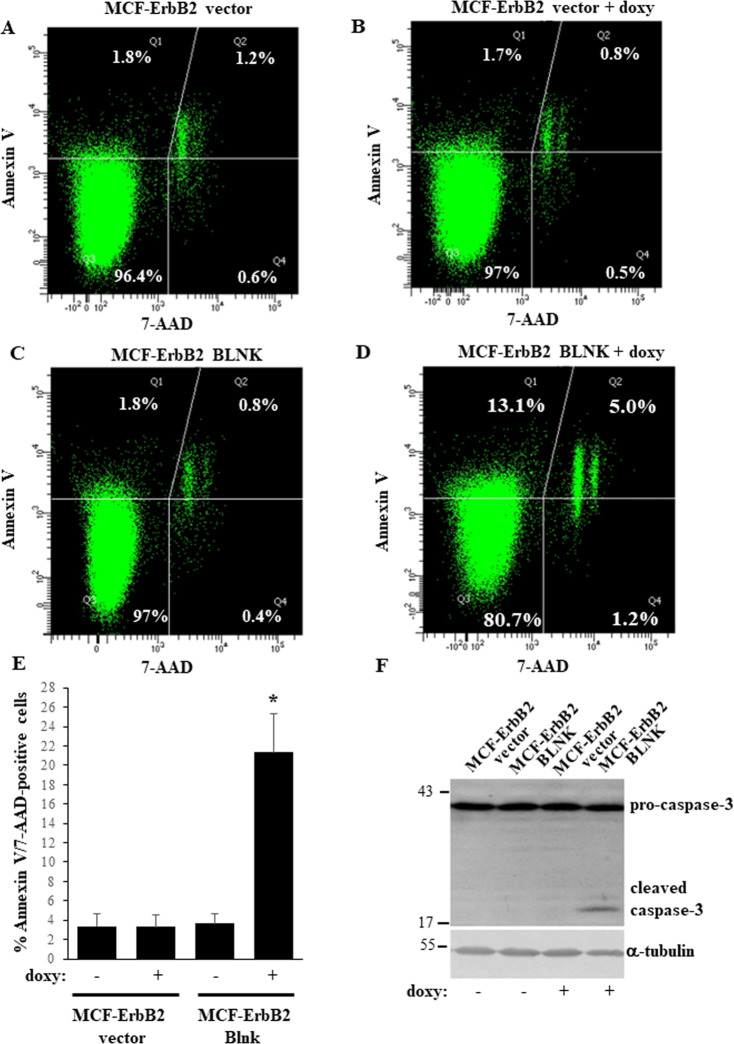
BLNK triggers apoptosis of detached ErbB2-overproducing breast cancer cells. **A**–**E** Indicated cell lines were cultured detached form the ECM in the absence (−) or in the presence (+) of 3 ng/ml doxycycline (doxy) for 24 h and assayed for Annexin V binding and permeability to 7-AAD by flow cytometry. Representative flow cytometry plots are shown in **A**–**D**. % Annexin V- and 7AAD-negative (lower left quadrant), Annexin V-positive and 7AAD-negative (upper left quadrant), Annexin V-positive and 7AAD-positive (upper right quadrant) and Annexin V-negative and 7AAD-positive (lower right quadrant) cells in the total cell population is shown. Results of three independent experiments plus SD are shown in (**E**). % Annexin V-positive/7-AAD positive cells in **E** is the sum of the percentages of Annexin V-positive/7AAD-negative, Annexin V-positive/7AAD-positive and Annexin V-negative/7AAD-positive cells. *p-value < 0.05. **F** Indicated cell lines were cultured detached form the ECM in the absence (−) or in the presence (+) of 3 ng/ml doxycycline (doxy) for 48 h and assayed for caspase-3 expression. α-tubulin was used as a loading control.

To examine the role of BLNK in the regulation of anoikis of ErbB2-overproducing breast cancer cells by a complementary approach, we generated a variant of MCF-ErbB2 cells MCF-ErbB2-BLNK in which exogenous BLNK expression is controlled by the doxycycline-inducible promoter (Fig. 4C). We found that treatment of these cells with doxycycline significantly upregulated BLNK compared to the control MCF-ErbB2-vector cells (Fig. 4C) or to the parental MCF-ErbB2 cells (Fig. 4D). Moreover, exogenous BLNK significantly reduced the number of detached MCF-ErbB2 cells (Fig. 4E). Unlike the case in culture, where the cells grow in the presence of significant amounts of growth factors and nutrients, cancer cells experience chronic metabolic stress in vivo due to disorganized tumor vascularization and the resulting insufficient growth factor and nutrient supply [24]. Notably, when we mimicked this stress by reducing the amount of serum and nutrients available to detached cells, the effect of BLNK on 3D growth of MCF-ErbB2 cells was significantly enhanced (Fig. 4F). BLNK-induced reduction in the number of detached cells observed by us was likely caused by cell death since it was accompanied by increased binding of detached BLNK-overproducing cells to Annexin V, an apoptosis symptom (Fig. 5A–E) [25]. Of note, apoptotic cells are not phagocytosed in culture and ultimately become permeable to vital dyes [26]. Indeed, we noticed that further to BLNK overexpression, some Annexin V-positive cells became permeable to a vital dye 7-AAD (Fig. 5A–D). In an effort to further demonstrate that BLNK triggers apoptosis of detached breast cancer cells we established that BLNK triggers the cleavage (a sign of activation) of procaspsase-3 (Fig. 5F), the key apoptosis executioner [27]. Thus, ErbB2-dependent BLNK downregulation is required for anoikis resistance of breast epithelial cells.

### p38MAP kinase mediates BLNK-dependent breast cancer cell anoikis

We further investigated the mechanisms of BLNK-dependent anoikis. It was proposed that simultaneous binding of distinct BLNK phospho-tyrosine residues to PLCγ and VAV, a GTP exchange factor for the Rho family GTPases, triggers a complex set of events that activate p38MAPK and a JNK protein kinases, both of which can kill cells [15]. Although all aspects of this model have not been formally proven, the ability of BLNK to activate p38MAPK and JNK is well-established [28, 29]. In addition, BLNK was proposed to be able to kill cells by binding a protein kinase JAK3 [23].

**Fig. 6:**
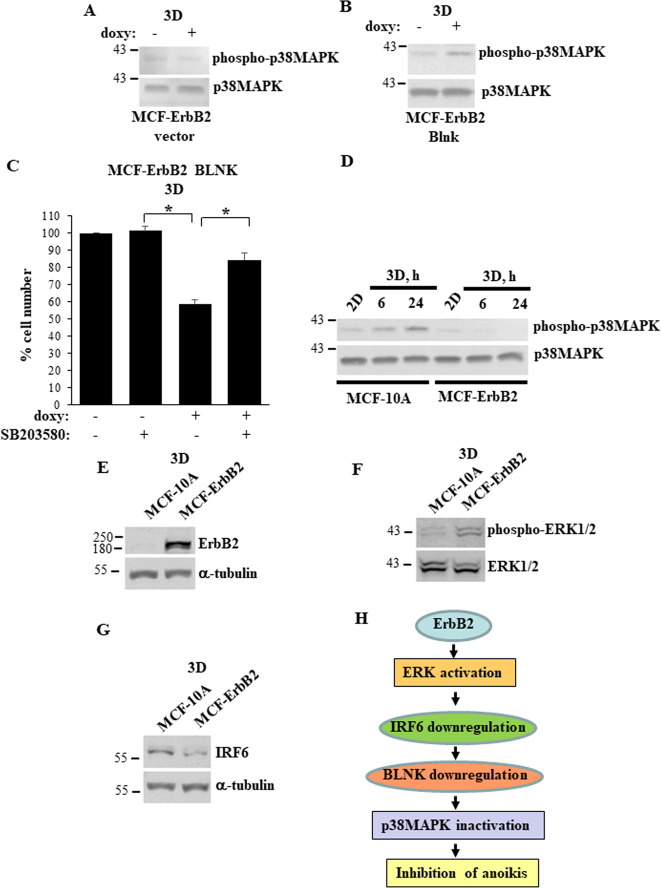
BLNK triggers anoikis of ErbB2-overproducing breast cancer cells by activating p38MAPK. **A**, **B** Indicated cell lines were cultured detached from the ECM (3D) for 54 h in the absence (−) or in the presence (+) of 3 ng/ml doxycycline (doxy) and assayed for phospho-p38MAPK and p38MAPK expression by western blotting. **C** MCF-ErbB2-BLNK cells were cultured as in **A**, **B** for 72 h in the absence (−) or in the presence (+) of 20 μM SB203580 and counted. The number of untreated cells was designated as 100%. The data are the average of the three independent experiments plus the SD. *p-value < 0.05. **D** MCF10A and MCF-ErbB2 cells were cultured attached to (2D) or detached from (3D) the ECM for the indicated times and assayed as in **A**, **B**. **A**–**G** Indicated cells were cultured detached from the ECM for 6 h and assayed for ErbB2 (**E**), phospho-Erk1/2 (**F**) and IRF6 (**G**) levels by western blotting. α-tubulin was used as a loading control in **E**, **G** and total ERK1/2, in **F**. **H** A model of ErbB2-dependent-inhibition of breast cancer cell anoikis that emerged from our study. ErbB2-dependent ERK activation downregulates IRF6, IRF6 downregulation causes reduction in the cellular BLNK levels, while BLNK loss in turn inactivates p38MAPK and thus blocks breast cancer cell anoikis.

We found that doxycycline-dependent BLNK upregulation in MCF-ErbB2-BLNK cells (see Fig. 4C) does not affect JNK phosphorylation (an established sign of JNK activation [28, 29]) (not shown). Moreover, we did not detect JAK3 expression in these cells (not shown). In contrast, we found that exogenous BLNK noticeably increases phosphorylation of p38MAPK, a symptom of p38MAPK activation [28, 29] (Fig. 6A, B). Four p38 MAPK isoforms are known, α, β, γ and δ. We found BLNK-dependent loss of MCF-ErbB2-BLNK cells in 3D culture is reversed by SB203580, a widely used small molecule inhibitor of p38MAPK α and β [30] (Fig. 6C).

ErbB2 prevents detachment-induced BLNK upregulation in breast epithelial cells (Fig. 1B). Since BLNK controls p38MAPK activity (Fig. 6A, B), detachment of non-malignant breast epithelial cells can be expected to activate p38MAPK and this activation can be anticipated to be blocked by ErbB2. This is, indeed, what we observed (Fig. 6D). We confirmed that similar to what we found previously [11], other elements of the BLNK-dependent anti-anoikis mechanism identified by us, i.e. ErbB2-dependent ERK activation and ErbB2-driven IRF6 downregulation, can be detected in our experimental setting (Fig. 6E–G). Thus, our data are consistent with a model whereby ErbB2-dependent ERK activation downregulates IRF6, IRF6 downregulation causes reduction in the cellular BLNK levels, whereas BLNK loss in turn inactivates p38MAPK and thus blocks breast cancer cell anoikis (Fig. 6H).

### Proteasome inhibition triggers BLNK-dependent death of detached breast cancer cells

**Fig. 7:**
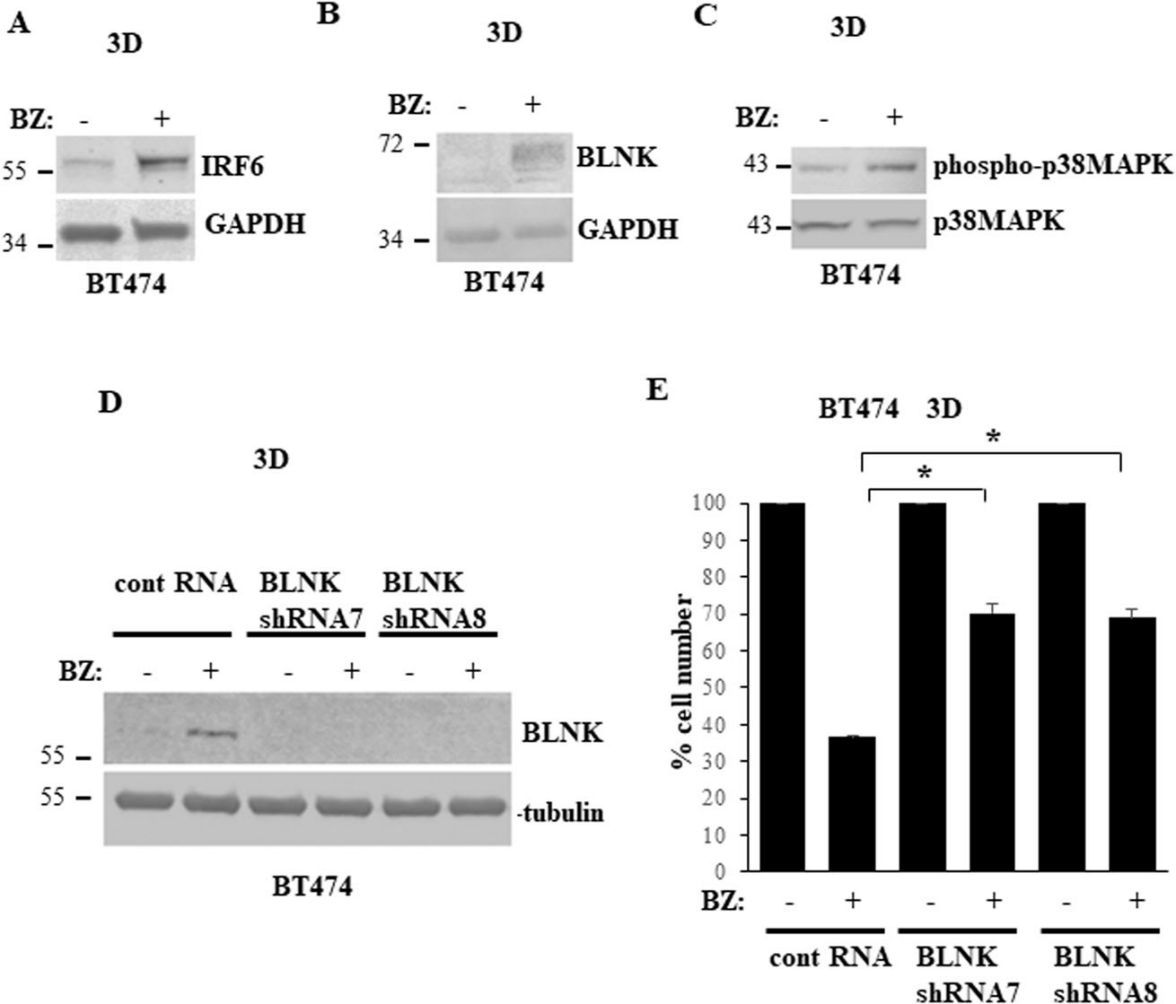
Bortezomib causes BLNK-dependent anoikis of ErbB2-overproducing breast cancer cells. **A**, **B** BT474 cells were cultured detached from the ECM (3D) for 48 h in the absence (−) or in the presence (+) of 100 nM bortezomib (BZ) and assayed for IRF6 (**A**), BLNK (**B**) and phospho-p38MAPK (**C**) expression by western blotting. GAPDH was used as a loading control in (A, B) and total p38MAPK, in **C**. **D** BT474 cells were infected with a control lentivirus or that encoding BLNK shRNA (BLNKshRNA) 7 or 8, cultured for 24 h in the absence (−) or in the presence (+) of 100 nM bortezomib (BZ) detached from the ECM (3D) and assayed for BLNK expression by western blotting. α-tubulin was used as a loading control. **E** BT474 cells were treated as in **C**, cultured detached from the ECM (3D) for 120 h in the absence (−) or in the presence (+) of 100 nM bortezomib (BZ) and counted. The number of control cells was designated as 100%. The data are the average of the three independent experiments plus the SD. *p-value < 0.05.

We further tested whether understanding of the mechanisms by which ErbB2 downregulates BLNK can potentially be used for designing anoikis-promoting breast cancer treatments. Others found that IRF6 protein turnover is controlled by the ubiquitin proteasome system [31]. Indeed, we observed that a small molecule proteasome inhibitor bortezomib used for multiple myeloma treatment [32] upregulates both IRF6 and its target BLNK in ErbB2-positive breast cancer cells BT474 (Fig. 7A, B). As expected, bortezomib treatment also increased phosphorylation of the BLNK effector p38MAPK (Fig. 7C). Furthermore, bortezomib triggered significant loss of detached BT474 cells and this loss was substantially reduced by BLNK knockdown by two separate shRNAs (Fig. 7D, E). Thus, proteasome inhibition represents potential novel pharmacological approach for causing BLNK-dependent breast cancer cell anoikis.

### BLNK blocks the ability of ErbB2-positive breast cancer cells to form tumors in vivo

**Fig. 8:**
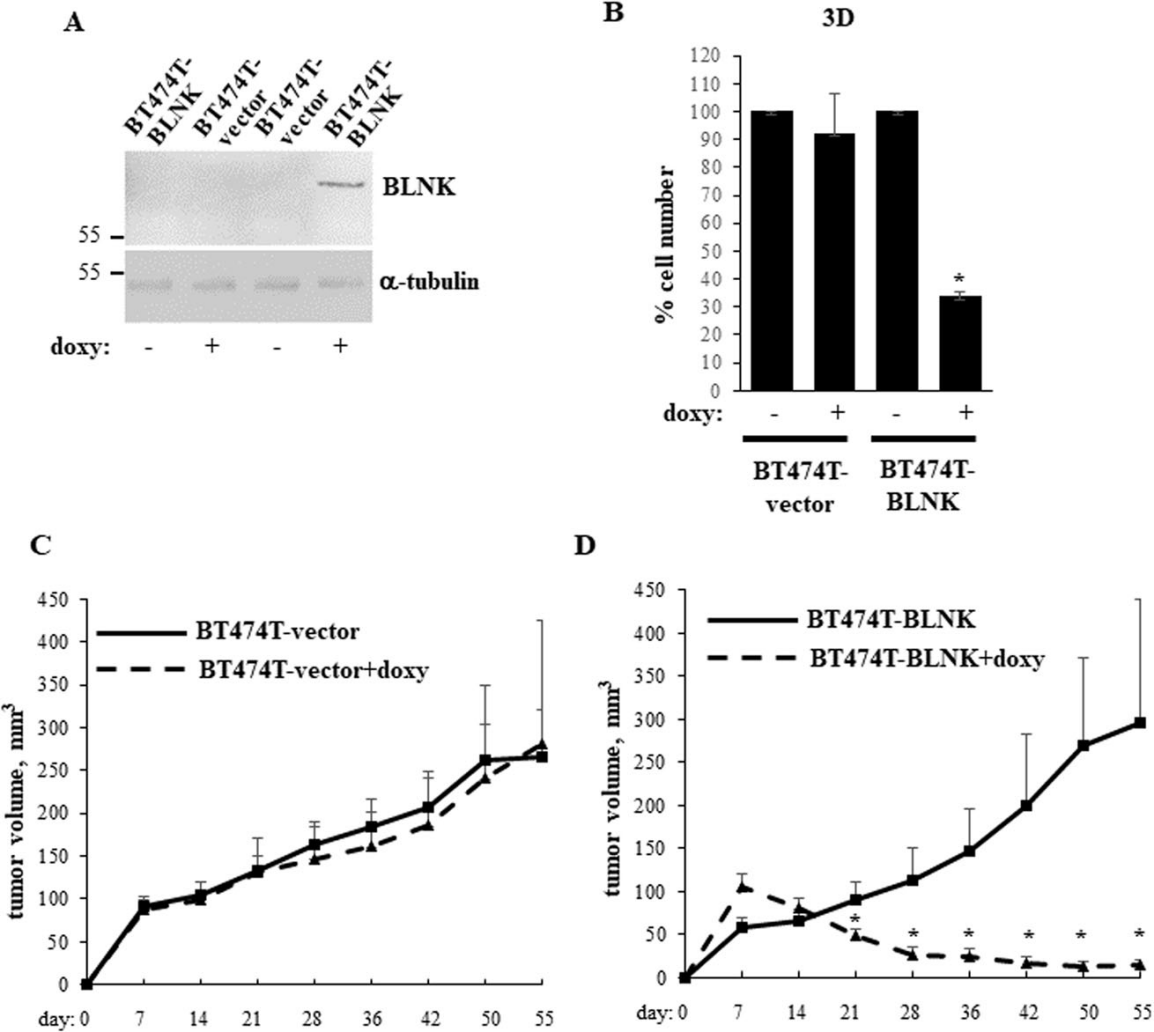
BLNK blocks the ability of ErbB2-positive breast cancer cells to form tumors in mice. **A** Indicated cell lines were cultured detached from the ECM (3D) for 24 h in the absence (−) or in the presence (+) of 500 ng/ml doxycycline (doxy) and assayed for BLNK expression by western blotting. α-tubulin was used as a loading control. **B** Indicated cell lines were cultured as in **A** for 120 h in the ″starvation″ medium which represented the Hybri-Care medium supplemented with 0.1% Fetal Bovine Serum and diluted 2.5-fold with HBSS medium and counted. The data are the average of the three independent experiments plus the SD. *p-value < 0.05. **C**, **D** Female Nu/Nu Nude mice were injected with the indicated cells in the mammary fat pad (*n* = 7). 2 mg/ml doxycycline was added or not to the animals’ drinking water. Tumor volumes were measured at the indicated times. The data represent the average tumor volumes plus the SE. One mouse injected with BTLN3-BLNK cells was sacrificed on day 36 due to weight loss, one mouse injected with BTLN3-BLNK cells and receiving doxycycline was sacrificed on day 42 due to a tumor ulcer, one mouse injected with BTLN3-vector cells and receiving doxycycline was sacrificed on day 36 and another one, on day 55 due to diarrhea.

To form 3D tumor masses, cancer cells need to resist anoikis [20, 33]. Thus, we tested whether BLNK upregulation blocks the ability of ErbB2-positive breast cancer cells to form tumors in mice. To this end, we used a variant of BT474 cells BT474T selected for increased tumorigenicity by serial passaging in mice upon injection in the mammary fat pad. We generated a variant of the cells BT474T-BLNK carrying the BLNK gene under the control of doxycycline-inducible promoter (Fig. 8A). Since as discussed above, tumor cells tend to grow under metabolic stress in vivo [24], we tested whether BLNK inhibits 3D growth of BT474T cells under such stress and found that this the case (Fig. 8B).

To examine the effect of BLNK on the in vivo tumorigenicity of these cells we injected the control or BT474T-BLNK cells in the mammary fat pads of immunodeficient mice receiving or not doxycycline. While the presence of doxycycline had no effect on tumorigenicity of the control cells (Fig. 8C), it strongly blocked tumorigenicity of BT474T-BLNK cells (Fig. 8D). Hence, BLNK upregulation blocks tumorigenicity of ErbB2-positive breast cancer cells in vivo.

### Increased BLNK breast tumor mRNA levels are associated with increased relapse-free survival of patients with ErbB2-positive breast cancer

**Fig. 9:**
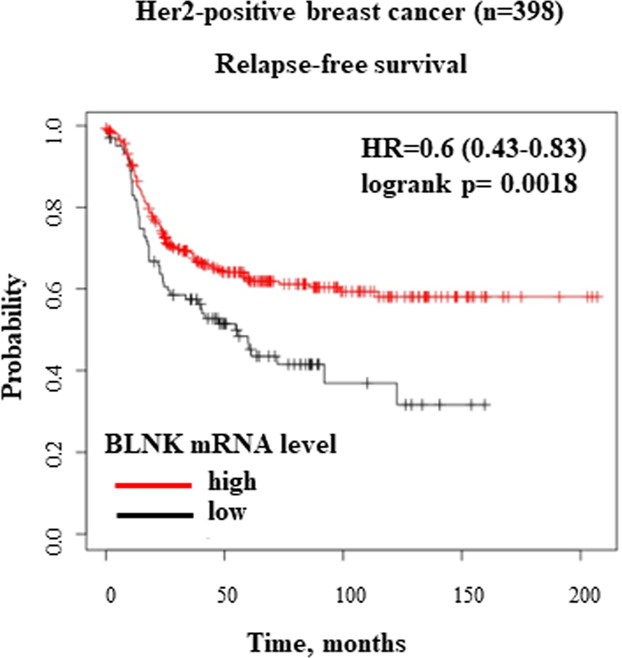
High BLNK mRNA levels in patients’ ErbB2-positive tumors are associated with increased relapse-free survival. Kaplan–Meier analysis-based estimation of probabilities of patients’ relapse-free survival depending on the level of BLNK mRNA was performed. Patients were split by ″lower quartile″, ER status - array was ″negative″, subtype PAM50 was ″HER2 + ″.

Since BLNK blocks breast cancer cell tumorigenicity (Fig. 8D), it is conceivable that increased BLNK gene expression in patients’ tumors is associated with slower breast cancer progression. To test whether this is the case we used Kaplan–Meier plotter, a web-based platform that allows the analysis of the gene microarray expression data on breast cancer-derived mRNAs based on the Gene Expression Omnibus and European Genome-Phenome Archive repositories [34]. We found that in a cohort of 398 patients with ErbB2/Her2-positive breast carcinoma, increased BLNK mRNA expression is significantly associated with increased disease-free survival: *p*-value = 0.0018, hazard ratio = 0.6 (95% CI 0.43–0.83) (Fig. 9). These data are consistent with our findings that BLNK blocks 3D breast cancer cell growth.

In summary, we have discovered a novel mechanism of ErbB2-driven breast tumor growth driven by ErbB2-dependent BLNK downregulation.

## Discussion

We found that ErbB2 promotes survival of breast cancer cell detached from the ECM by a novel mechanism involving downregulation of transcription factor IRF6 and further downregulation of the cell death-promoting protein BLNK [23, 35]. ErbB2-induced BLNK downregulation likely blocks the apoptotic form of death of detached cancer cells since we observed that ectopic BLNK triggers apoptosis symptoms in the cells, such as caspase-3 cleavage and Annexin V positivity (Fig. 5). We found previously that these changes when observed in a fraction of cells at discreet time points, tend to accumulate in the total cell population over time and ultimately, cause significant loss of detached breast cancer cells [8].

We also found that ErbB2-induced BLNK downregulation inactivates a BLNK effector p38MAPK [15]. These data are consistent with findings that p38MAPK can kill cells by phosphorylating and inactivating the anti-apoptotic proteins BCL2, MCL1 and BCLX_L_ [36] and/or phosphorylating and activating the pro-apoptotic proteins BAX and BIM [36]. Moreover, we [37] and others [38] found that detachment-induced p38MAPK activation triggers anoikis of non-malignant intestinal and breast epithelial cells respectively.

We found that BLNK upregulation in breast tumor cells by a genetic approach blocks their tumorigenicity (Fig. 8). We also observed that a small molecule proteasome inhibitor bortezomib used for multiple myeloma treatment upregulates IRF6 and BLNK in detached ErbB2-positive breast cancer cells and kills them in a BLNK-dependent manner (Fig. 7). These data are consistent with the known ability of bortezomib to strongly suppress viability of ErbB2-positive breast cancer cells, including BT474 cells used in this study, by triggering their apoptosis [39, 40]. Our data indicate that proteasome inhibition represents a potential pharmacological approach for upregulating BLNK in breast cancer cells. This approach could serve for enhancing the anti-tumor effect of ErbB2-targeted therapeutic agents or for treatment of breast cancers resistant to these drugs. Bortezomib is presently being tested as an anti-cancer drug in a breast cancer clinical trial (ClinicalTrials.gov identifier: NCT04265872) and several trials directed at other solid tumors types (see ClinicalTrial.gov). Moreover, novel proteasome inhibitors are now being developed for solid tumor treatment [41] and could be explored as BLNK-upregulating drugs in ErbB2-positive breast cancer.

Another possible application of our finings is to use BLNK as a predictive marker in ErbB2-positive breast cancer. Conceivably, the ability of ErbB2 to downregulate BLNK depends on the degree of ErbB2 upregulation in the tumor. Moreover, ErbB2-positive tumors contain numerous mutations that differ between patients [42] and could also affect tumor BLNK levels and disease progression. We found that increased BLNK mRNA expression is significantly associated with increased disease-free survival of patients with ErbB2/Her2-positive breast carcinoma (Fig. 9). Utilizing such data to predict whether the patient is likely to benefit from the intended therapies could allow oncologists to modify these therapies earlier than is presently possible to better manage the disease.

## Supplementary information

Supplementary figures

Supplementary table 1

Original western blots

Reproducibility checklist

## Data Availability

The datasets used for assessment of probability of patient’s relapse-free survival are publicly available and were analyzed using the Kaplan–Meier plotter [34].
