# Supplementary material for: ErbB2/Her2-dependent downregulation of a cell death-promoting protein BLNK in breast cancer cells is required for 3D breast tumor growth
Source: Cell Death Dis. 2022 Aug 6;13(8):687. doi: 10.1038/s41419-022-05117-9 (PMC9357009; doi:10.1038/s41419-022-05117-9)
Supplement: Supplementary file 1 — Supplementary figures [file 41419_2022_5117_MOESM1_ESM.docx]

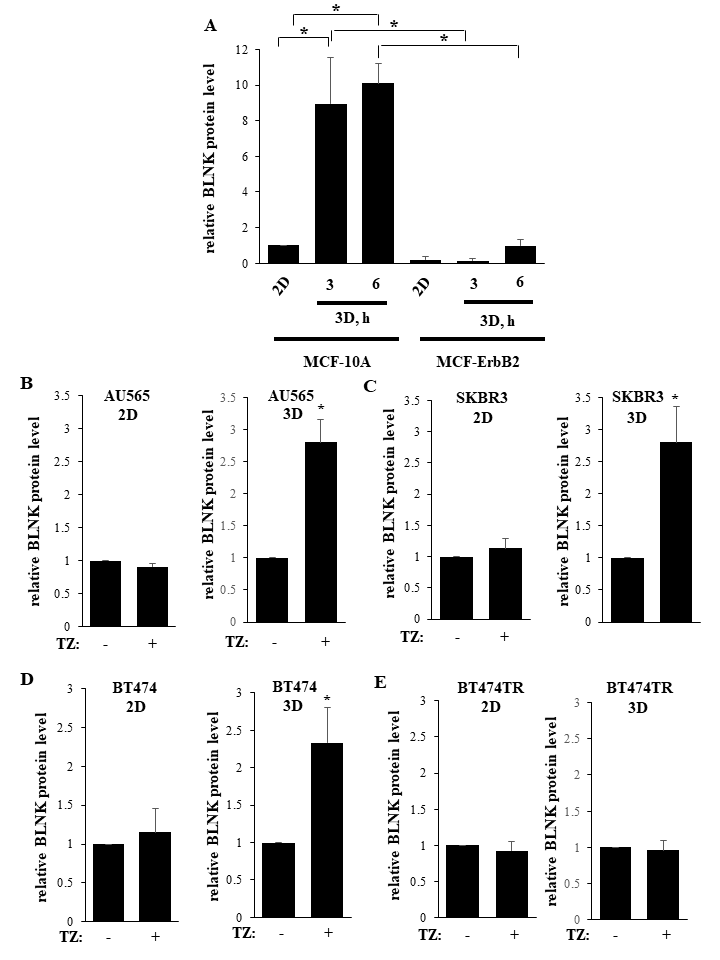


Supplementary fig. 1 Quantification of the western blots shown in Fig. 1. The data in (A) represent quantification of the data shown in Fig. 1A, the data in (B-E) represent quantification of the data shown in Fig. 1(C-F), respectively. The data represent the average of three independent experiments plus the SD. BLNK protein levels were normalized by those of the respective loading control in the case of each experiment. * - p˂0.05.


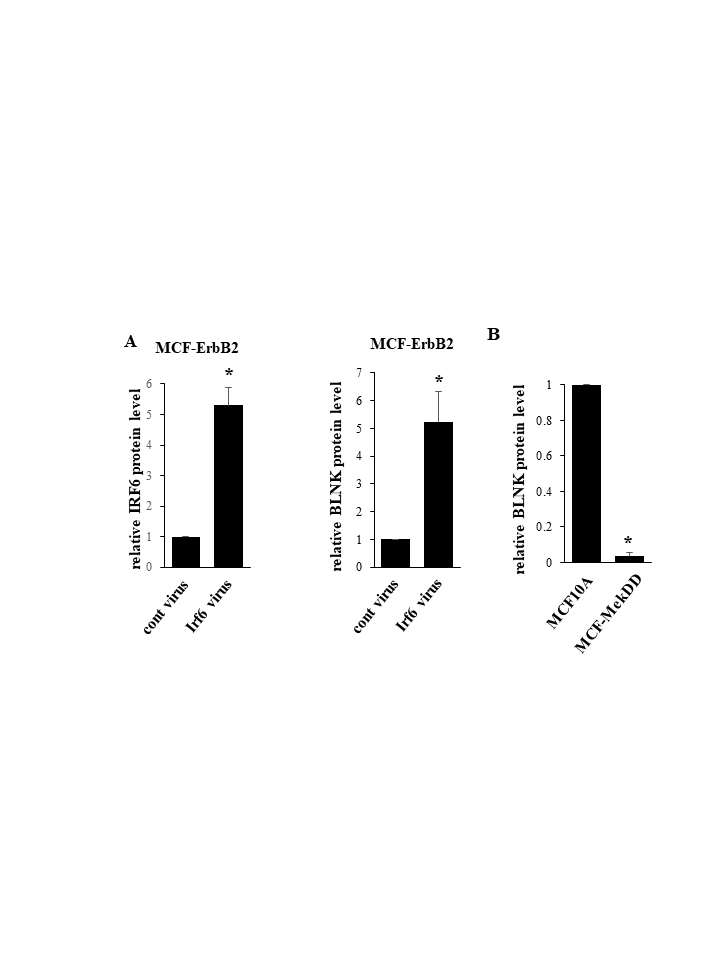
Supplementary fig. 2 Quantification of the western blots shown in Fig. 2. The data in (A) represent quantification of the data shown in Fig. 2A, the data in (B) represent quantification of the data shown in Fig. 2B. The data represent the average of three independent experiments plus the SD. IRF6 and BLNK protein levels were normalized by those of the respective loading control in the case of each experiment. * - p˂0.05.


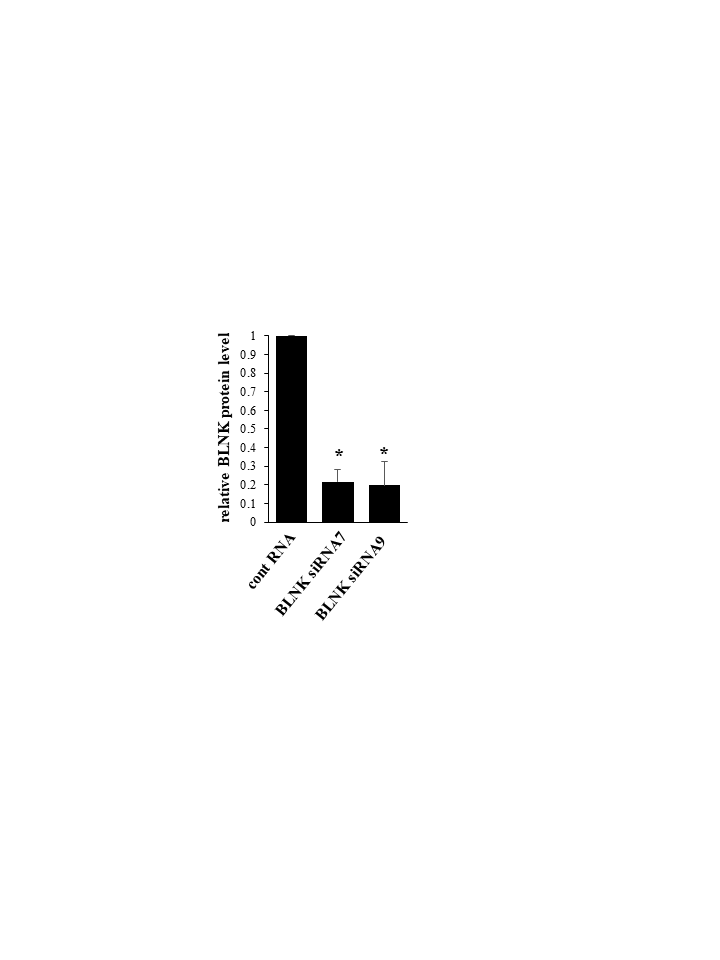


Supplementary fig. 3 Quantification of the western blot shown in Fig. 3A. The data represent the average of three independent experiments plus the SD. BLNK protein levels were normalized by those of the respective loading control in the case of each experiment. * - p˂0.05.


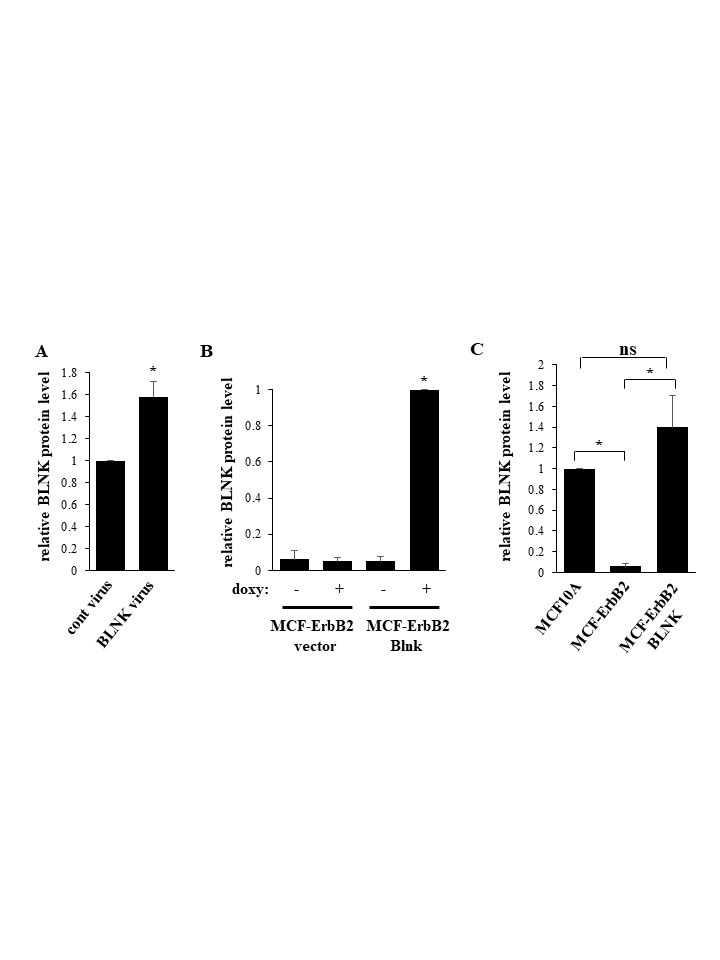


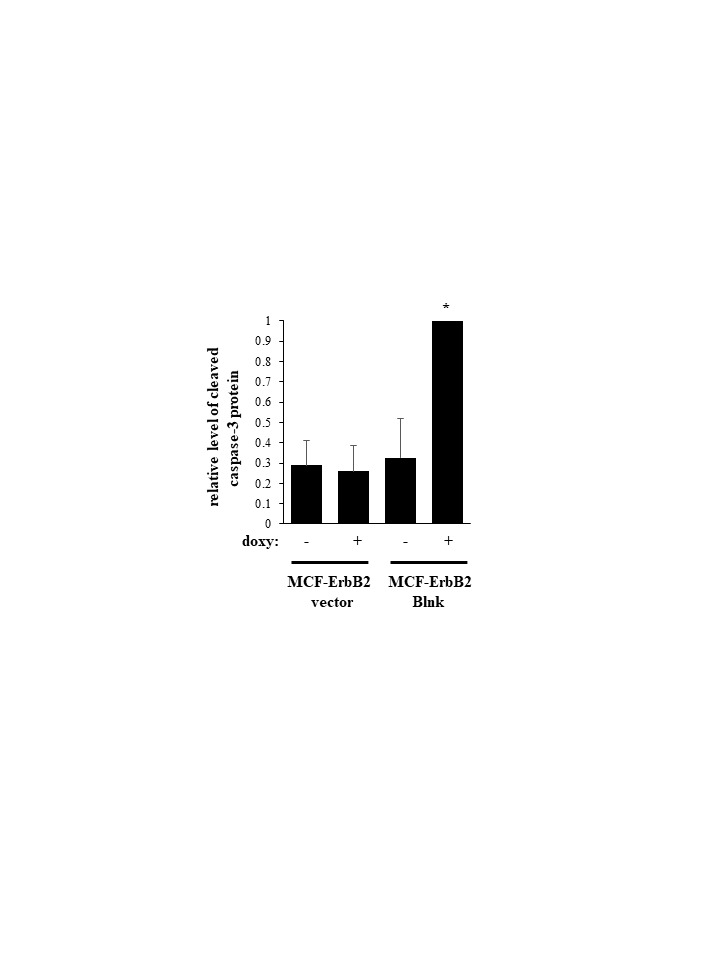
Supplementary fig. 4 Quantification of the western blots shown in Fig. 4. The data in (A) represent quantification of the data shown in Fig. 4A, the data in (B, C) represent quantification of the data shown in Fig. 4C, D, respectively. The data in (C) represent the average of two independent experiments plus the SD. The data in (A, B) represent the average of three independent experiments plus the SD. BLNK protein levels were normalized by those of the respective loading control in the case of each experiment. ns – not significant, * - p˂0.05.

Supplementary fig. 5 Quantification of the western blot shown in Fig. 5F. The data represent the average of three independent experiments plus the SE. cleaved caspase-3 protein levels were normalized by those of the respective loading control in the case of each experiment. * - p˂0.05.


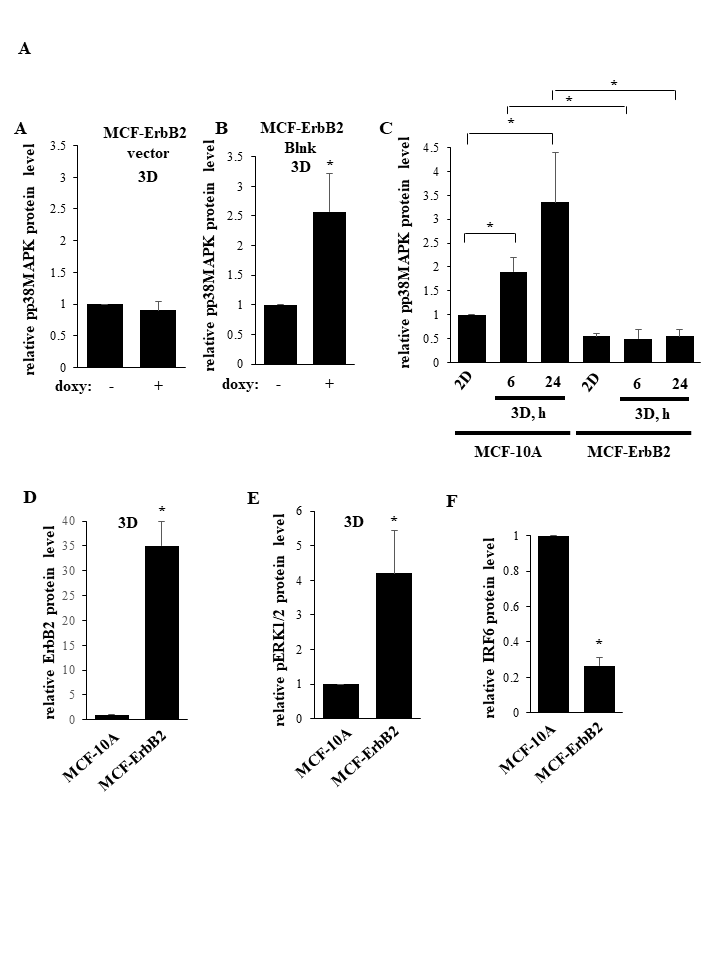
Supplementary fig. 6 Quantification of the western blots shown in Fig. 6. The data in (A, B) represent quantification of the data shown in Fig. 6A, B, respectively, the data in (C-F) represent quantification of the data shown in Fig. 6 (D-G), respectively. The data in (C) represent the average of two independent experiments plus the SD. The data in (A, B, D-F) represent the average of three independent experiments plus the SD. BLNK, pp38MAPK, ErbB2, pERK1/2 and IRF6 protein levels were normalized by those of the respective loading control in the case of each experiment. * - p˂0.05.


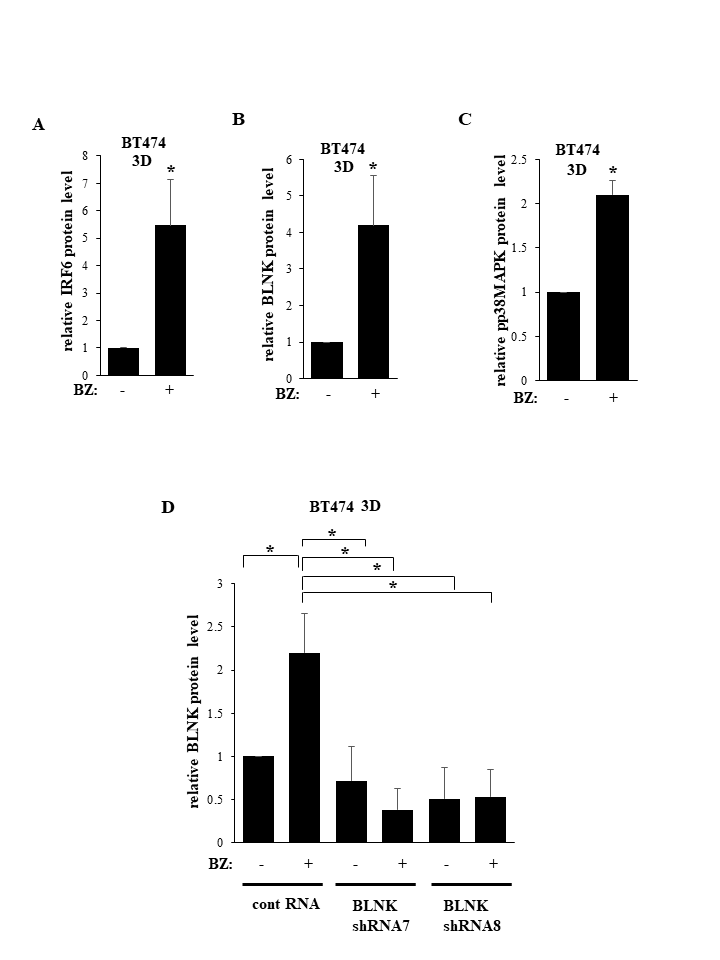
Supplementary fig. 7 Quantification of the western blots shown in Fig. 7. The data in (A-D) represent quantification of the data shown in Fig. 7(A-D), respectively. The data represent the average of three independent experiments plus the SD. IRF6, BLNK and pp38MAPK protein levels were normalized by those of the respective loading control in the case of each experiment. * - p˂0.05.


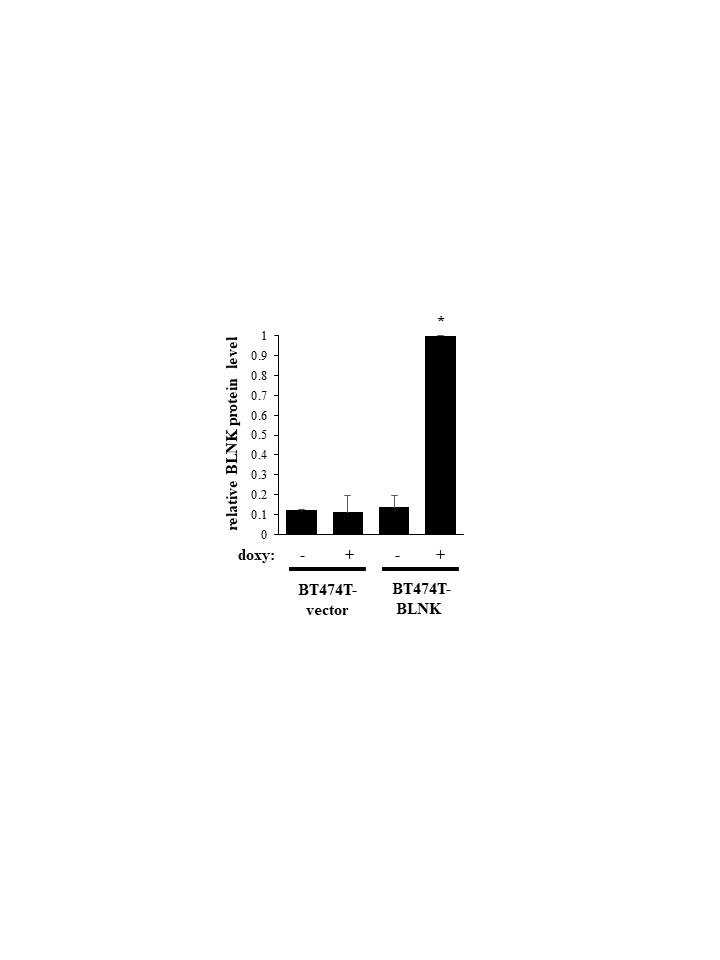


Supplementary fig. 8 Quantification of the western blot shown in Fig. 8A. The data represent the average of two independent experiment plus SD. BLNK protein levels were normalized by those of the respective loading control in the case of each experiment. * - p˂0.05
