# Supplementary material for: ErbB2/Her2-dependent downregulation of a cell death-promoting protein BLNK in breast cancer cells is required for 3D breast tumor growth
Source: Cell Death Dis. 2022 Aug 6;13(8):687. doi: 10.1038/s41419-022-05117-9 (PMC9357009; doi:10.1038/s41419-022-05117-9)
Supplement: Supplementary file 2 — Supplementary table 1 [file 41419_2022_5117_MOESM2_ESM.docx]

BLNK siRNA7 UAAUAUUCCUGUGCGAUUU

BLNK siRNA9 GCAAGACACUUCCCAGUAA

BLNKshRNA7 CCCATACCTCTGCCAAGATTT

BLNKshRNA8 CCATGATTCCAAACAACCATA

BLNK forward primer GAGGAGCAGTGGTCCGATGACT

BLNK reverse primer GGAATGCCTCTGGCTTGATCGA

18S rRNA forward primer ATAGTCAAGTTCGACCGTCTTC

18S rRNA reverse primer GTTGATTAAGTCCCTGCCCTT

**Supplementary table 1** Sequences of siRNAs, shRNAs and DNA primers used in the study.
