# Supplementary material for: ErbB2/Her2-dependent downregulation of a cell death-promoting protein BLNK in breast cancer cells is required for 3D breast tumor growth
Source: Cell Death Dis. 2022 Aug 6;13(8):687. doi: 10.1038/s41419-022-05117-9 (PMC9357009; doi:10.1038/s41419-022-05117-9)
Supplement: Supplementary file 3 — Original western blots [file 41419_2022_5117_MOESM3_ESM.docx]

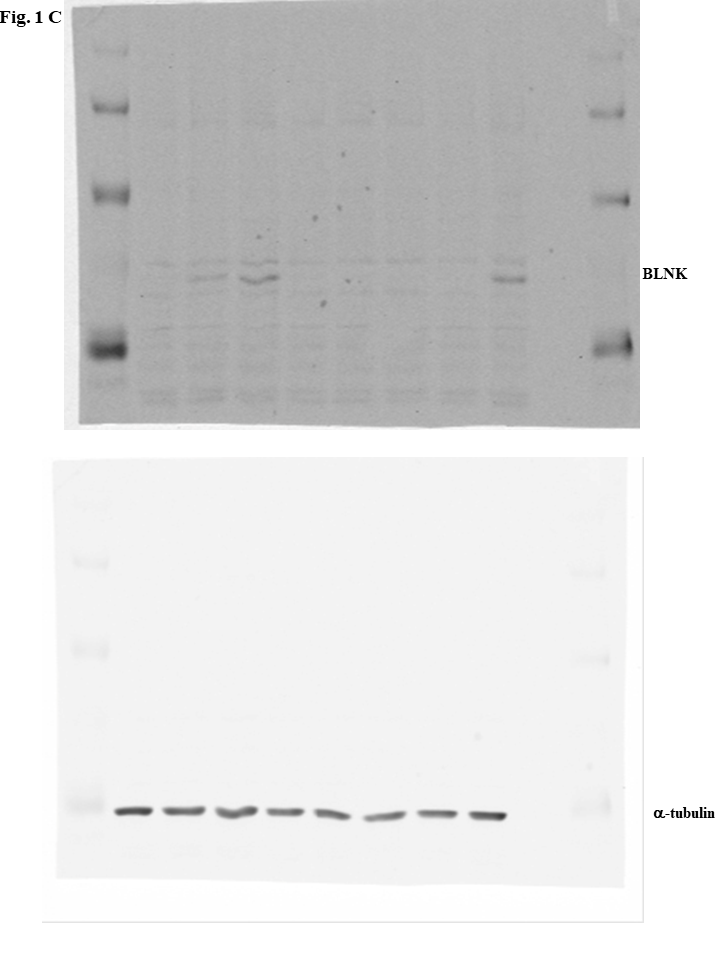


Supplementary fig. 9 Original western blots for the data shown in Fig. 1B. Lanes 1-6 are shown

in Fig. 1B.


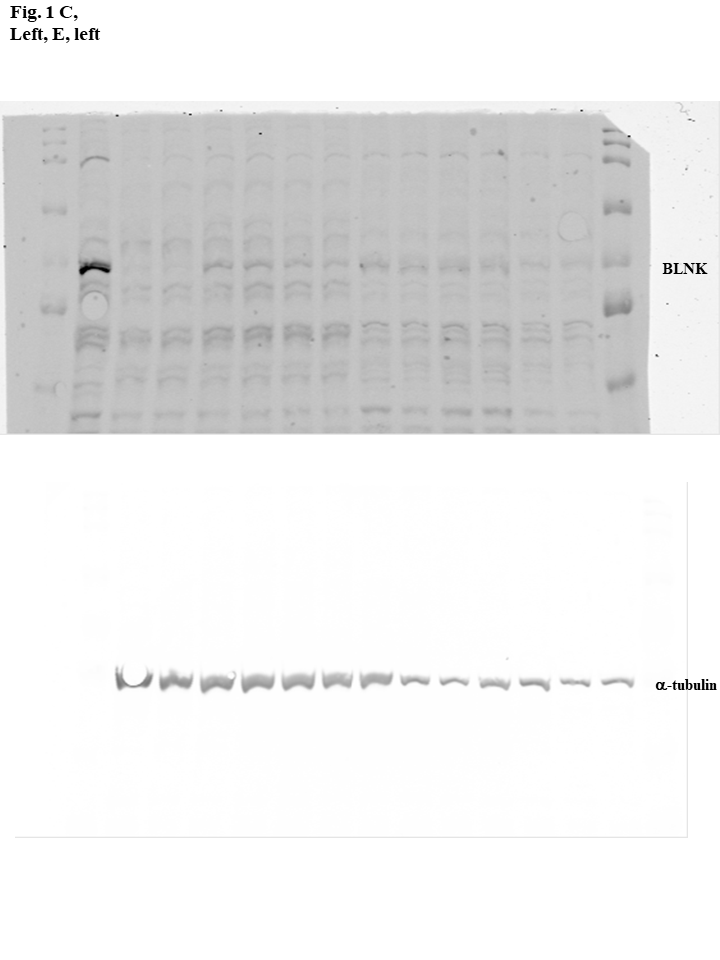


Supplementary fig. 10 Original western blots for the data shown in Fig. 1C, left, Fig. E, left. Lanes 3, 4 are shown in Fig. 1C, left and lanes 10, 11, in Fig. 1E, left


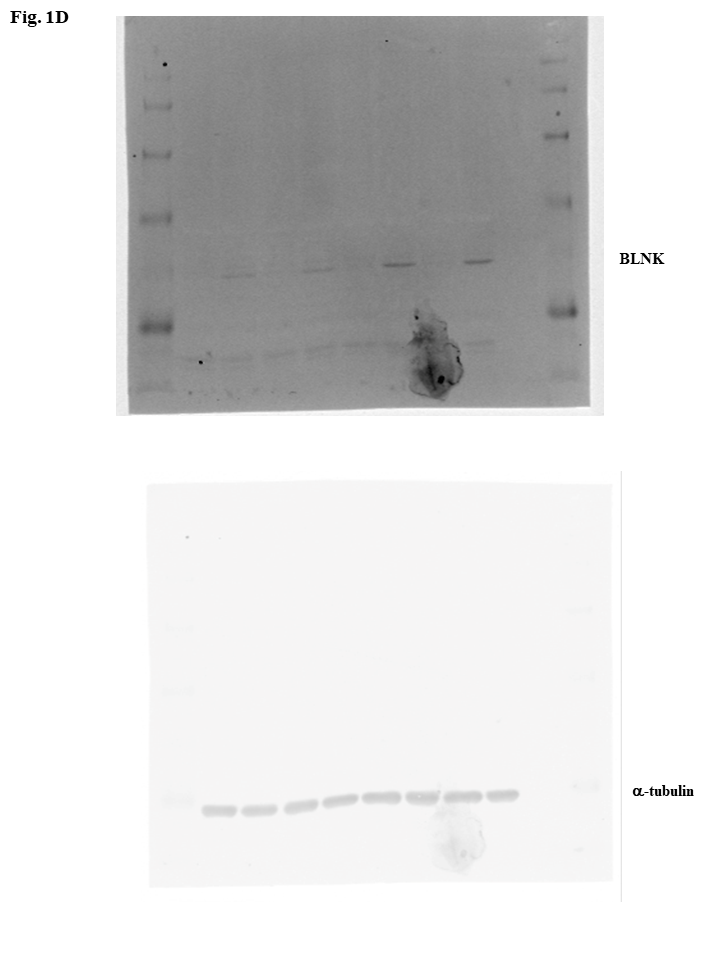


Supplementary fig. 11 Original western blots for the data shown in Fig. 1C, right and Fig. 1D, right. Lanes 5, 6 are shown in Fig. 1C, right and lanes 7, 8 are shown in Fig. 1D, right.


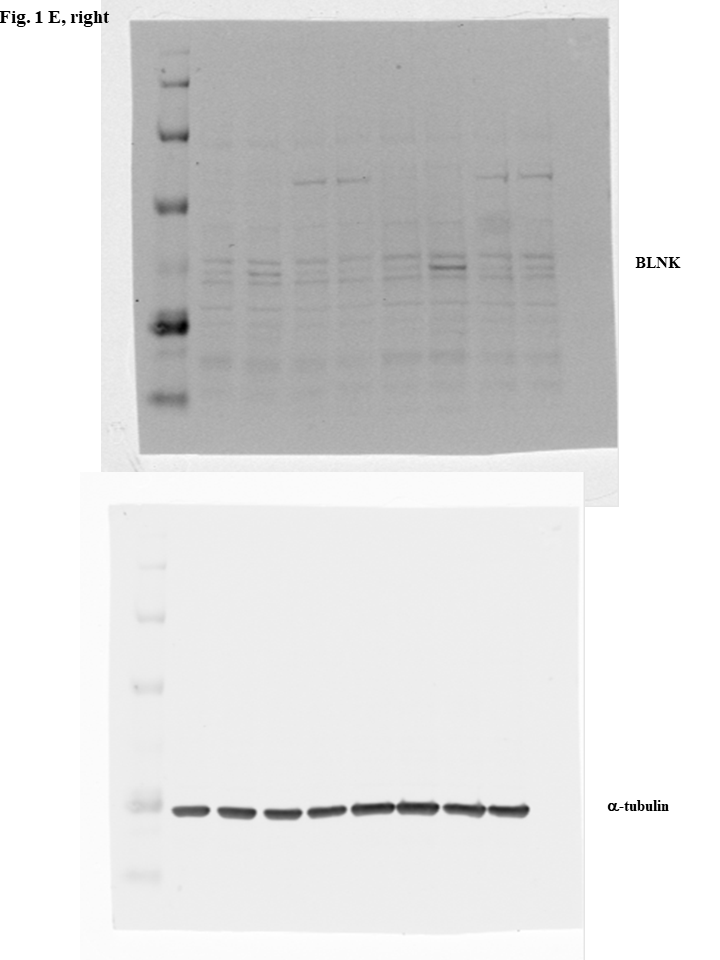


Supplementary fig. 12 Original western blots for the data shown in Fig. 1E, right. Lanes 1-4 are shown in Fig. 1E, right.


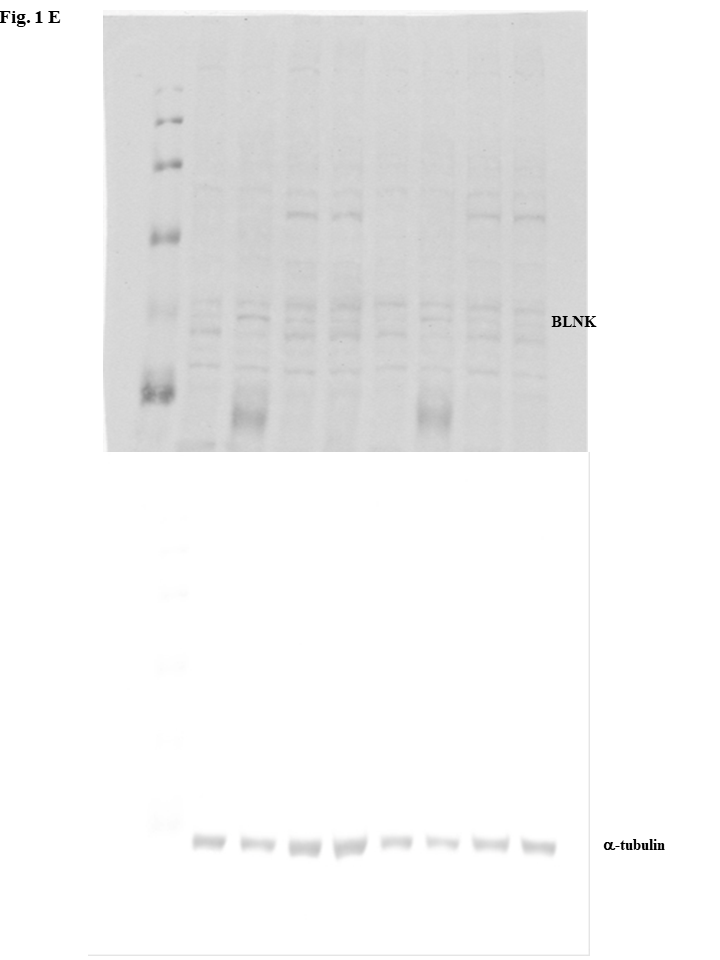


Supplementary fig. 13 Original western blots for the data shown in Fig. 1F, right. Lanes 3, 4 are shown in Fig. 1F, right.


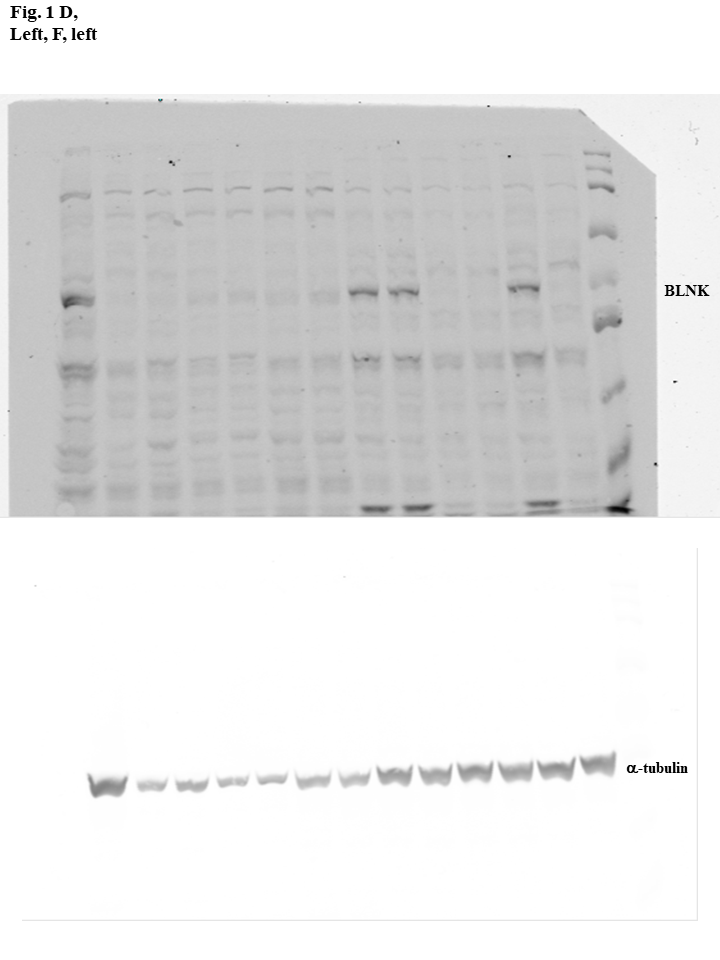


Supplementary fig. 14 Original western blots for the data shown in Fig. 1D, left and 1F, left. Lanes 10, 11 are shown in Fig. 1D, left and lanes 4, 5 are shown in Fig. 1F, left.


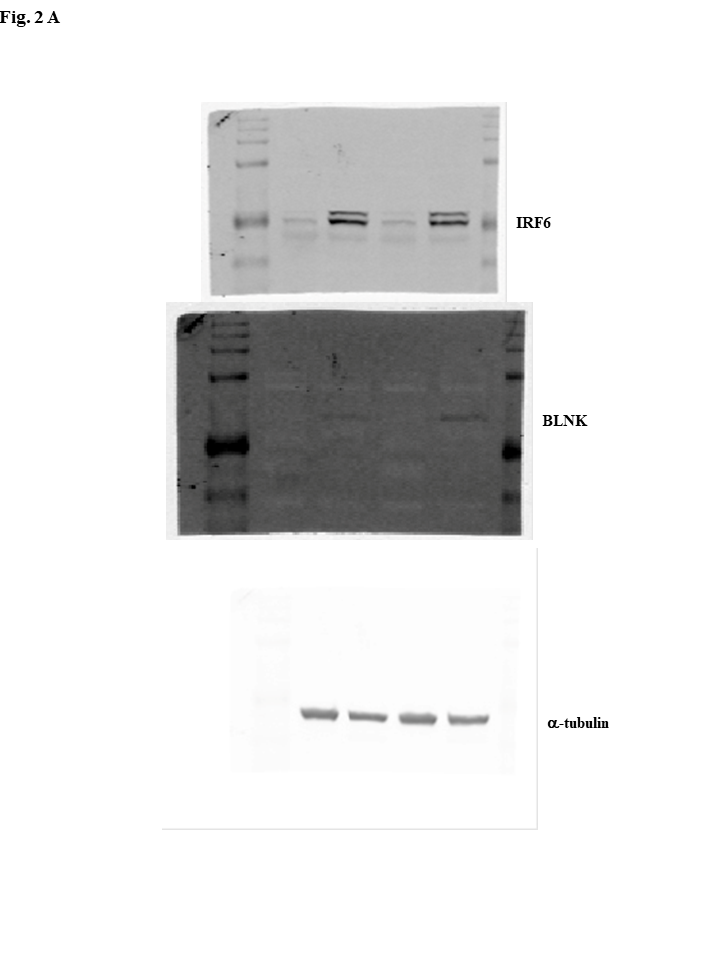


Supplementary fig. 15 Original western blots for the data shown in Fig. 2A. Lanes 3, 4 are shown in Fig. 2A.


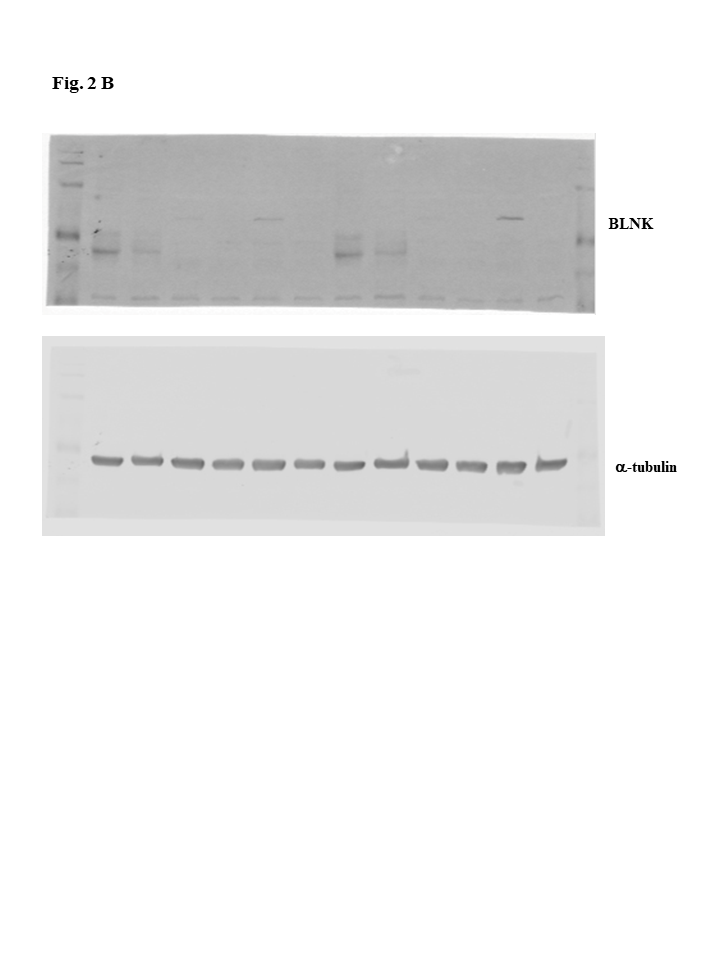


Supplementary fig. 16 Original western blots for the data shown in Fig. 2B. Lanes 11, 12 are shown in Fig. 2B.


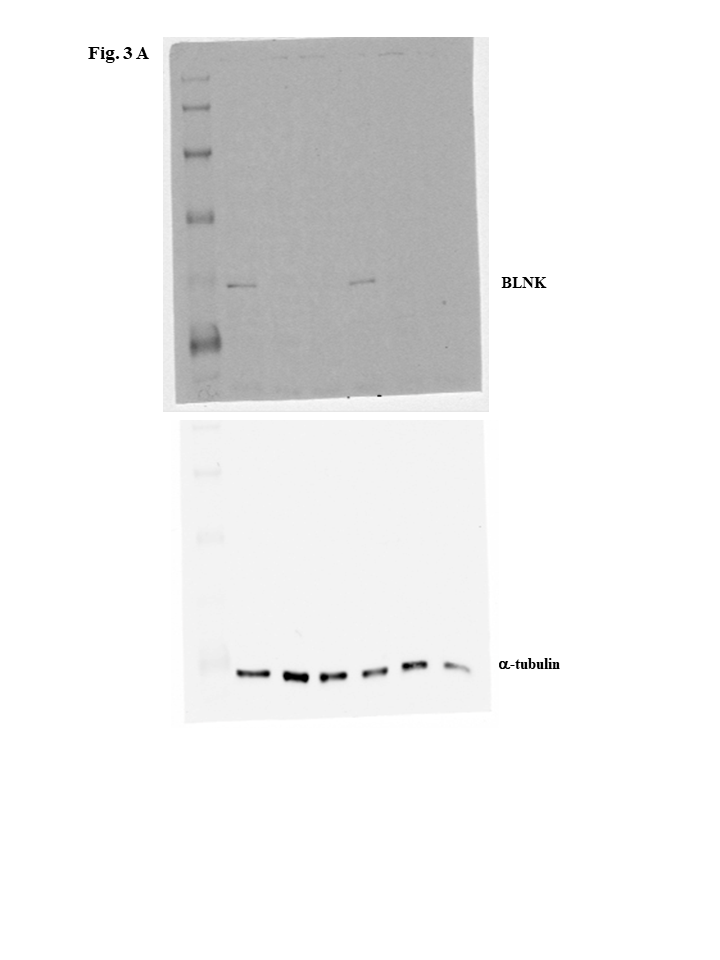
Supplementary fig. 17 Original western blots for the data shown in Fig. 3A. Lanes 1-3 are shown in Fig. 3A.


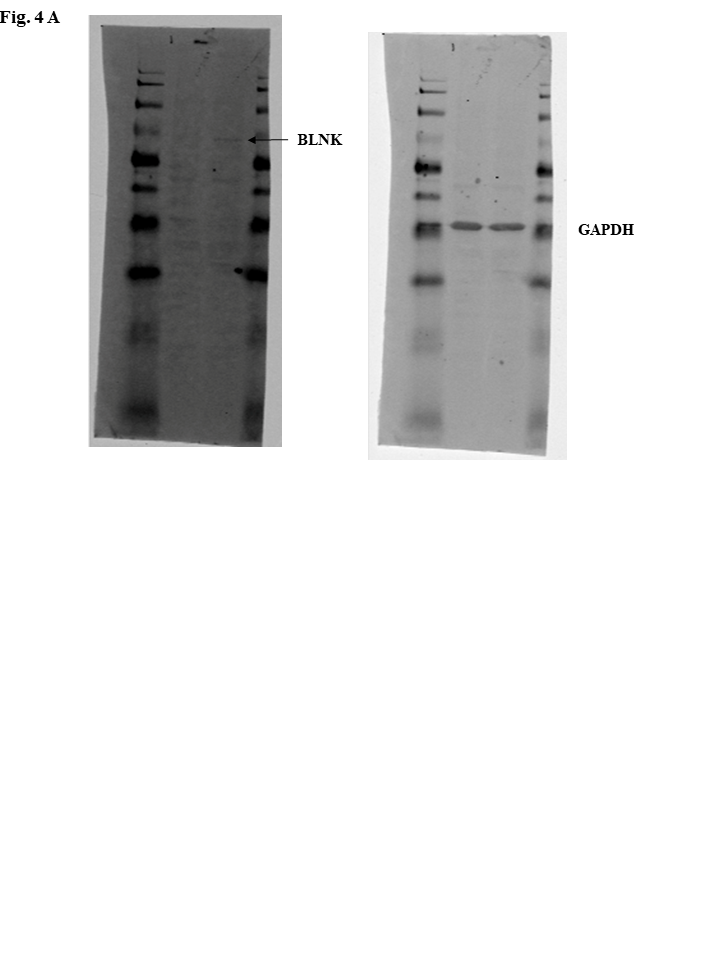
Supplementary fig. 18 Original western blots for the data shown in Fig. 4A


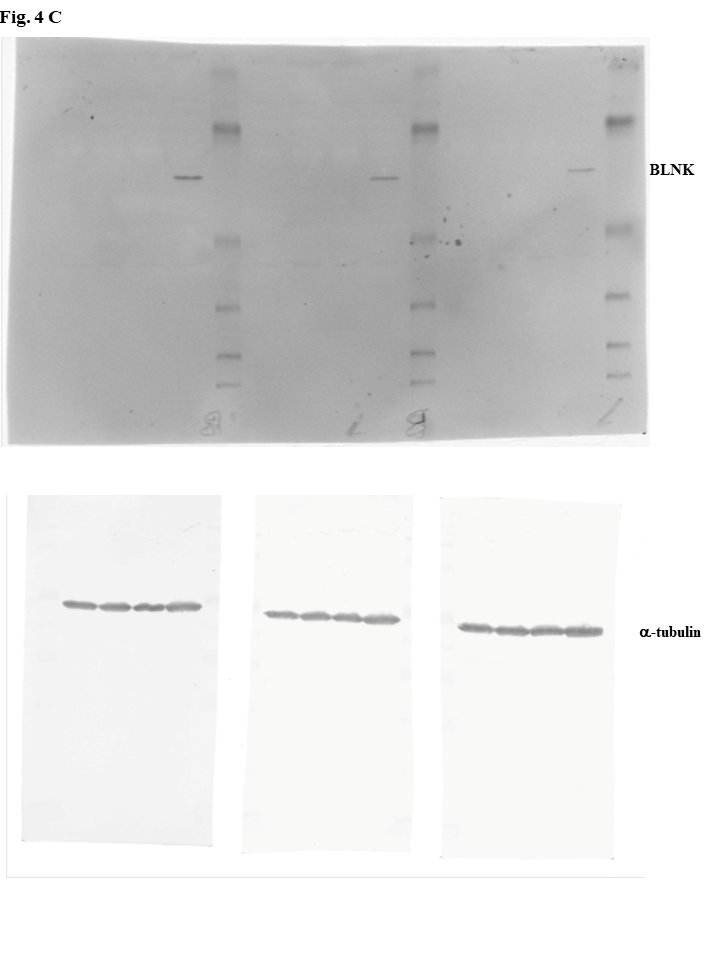


Supplementary fig. 19 Original western blots for the data shown in Fig. 4C. Lanes 1-4 are shown in Fig. 4C.


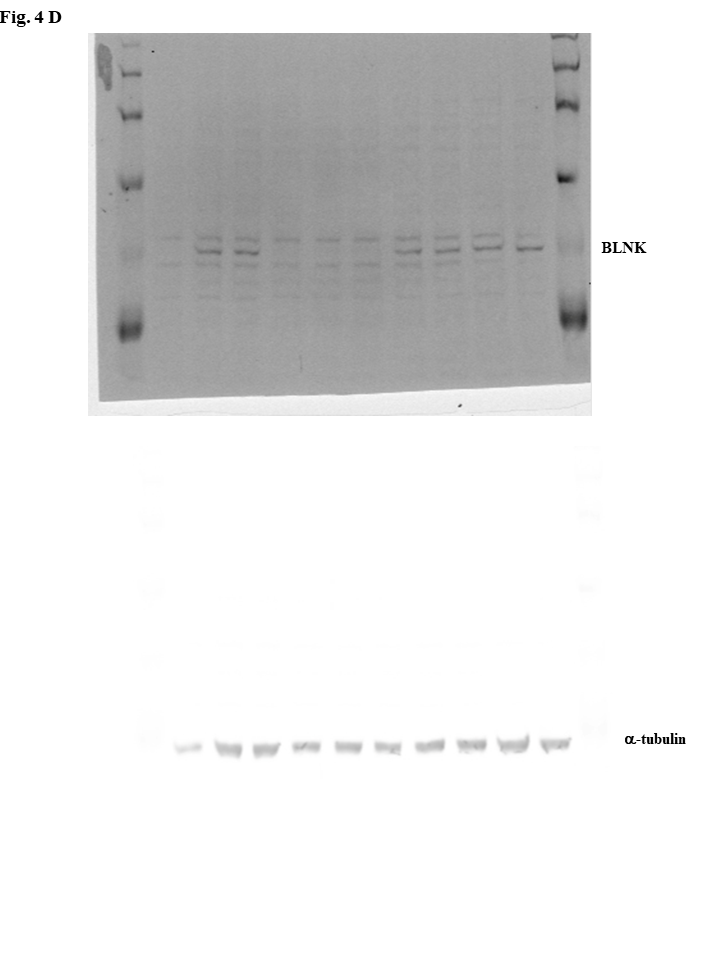


Supplementary fig. 20 Original western blots for the data shown in Fig. 4D. Lanes 3, 6, 10 are shown in Fig. 4D.


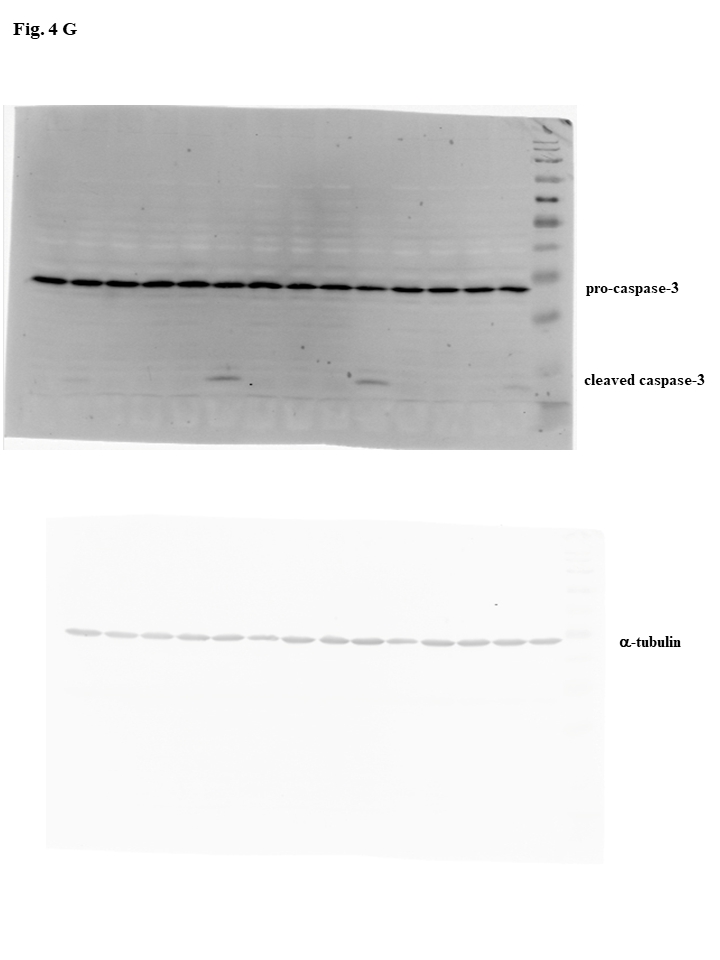


Supplementary fig. 21 Original western blots for the data shown in Fig. 5F. Lanes 3-6 are shown in Fig. 4G.


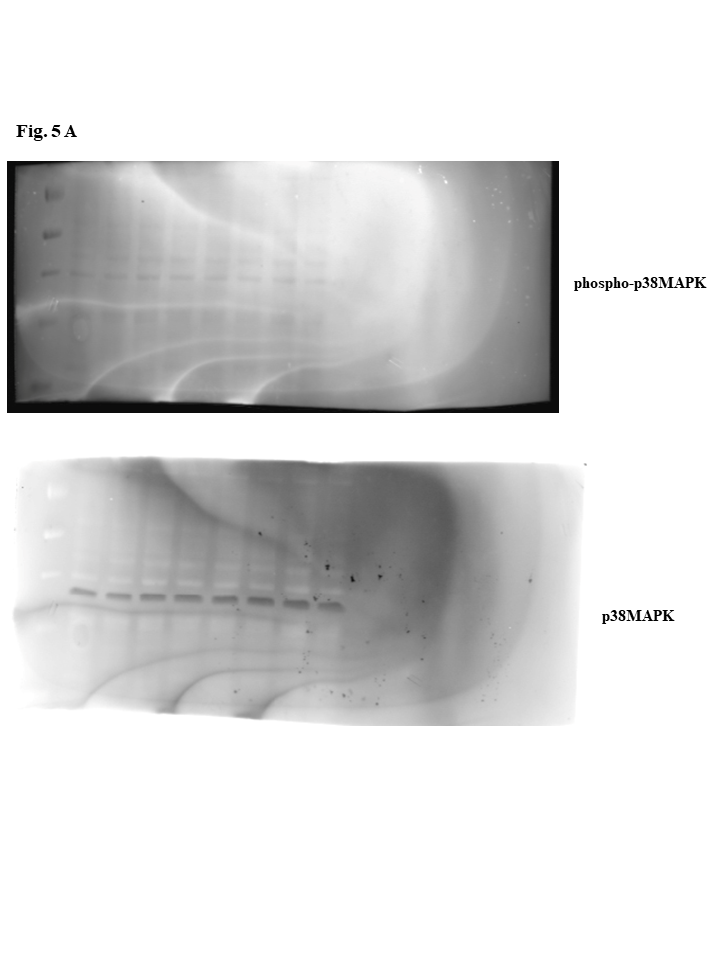


Supplementary fig. 22 Original western blots for the data shown in Fig. 6A. Lanes 3, 4 are shown in Fig. 6A.


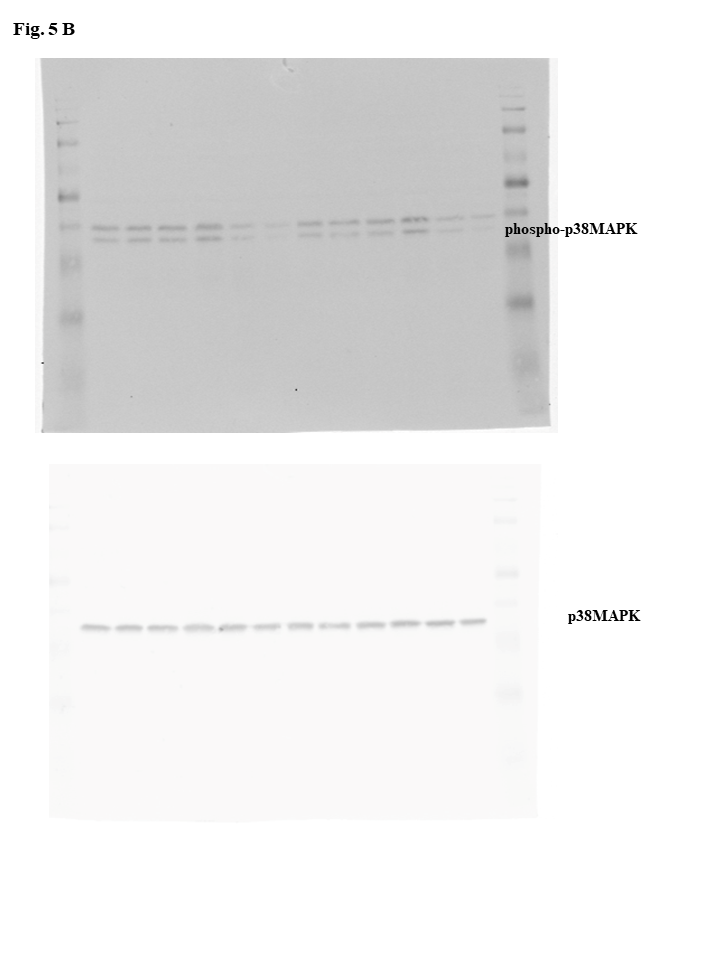


Supplementary fig. 23 Original western blots for the data shown in Fig. 6B. Lanes 9, 10 are shown in Fig. 6B.


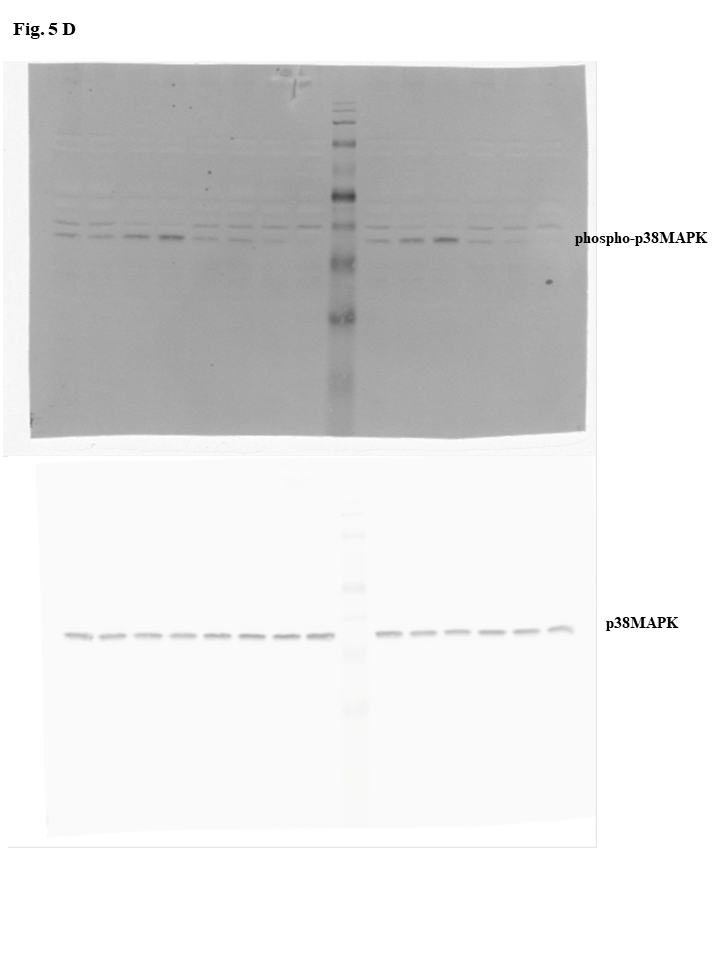
Supplementary fig. 24 Original western blots for the data shown in Fig. 6D. Lanes 10-15 are shown in Fig. 6D.


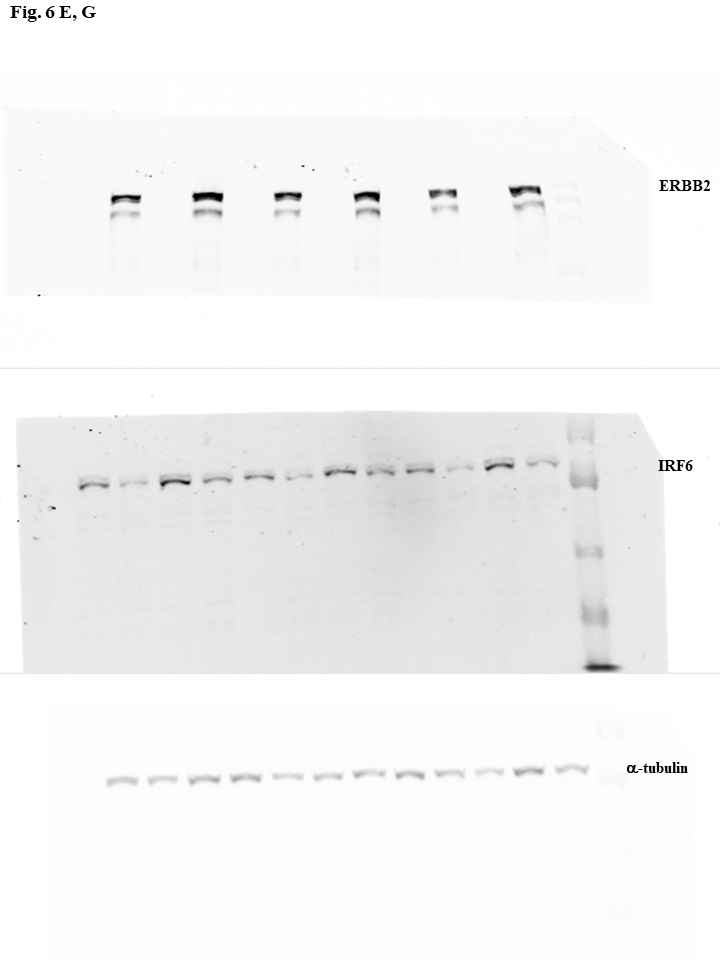


Supplementary fig. 25 Original western blots for the data shown in Fig. 6E, G. Lanes 1, 2 are shown in Fig. 6E and lanes 5, 6 are shown in Fig 6G.


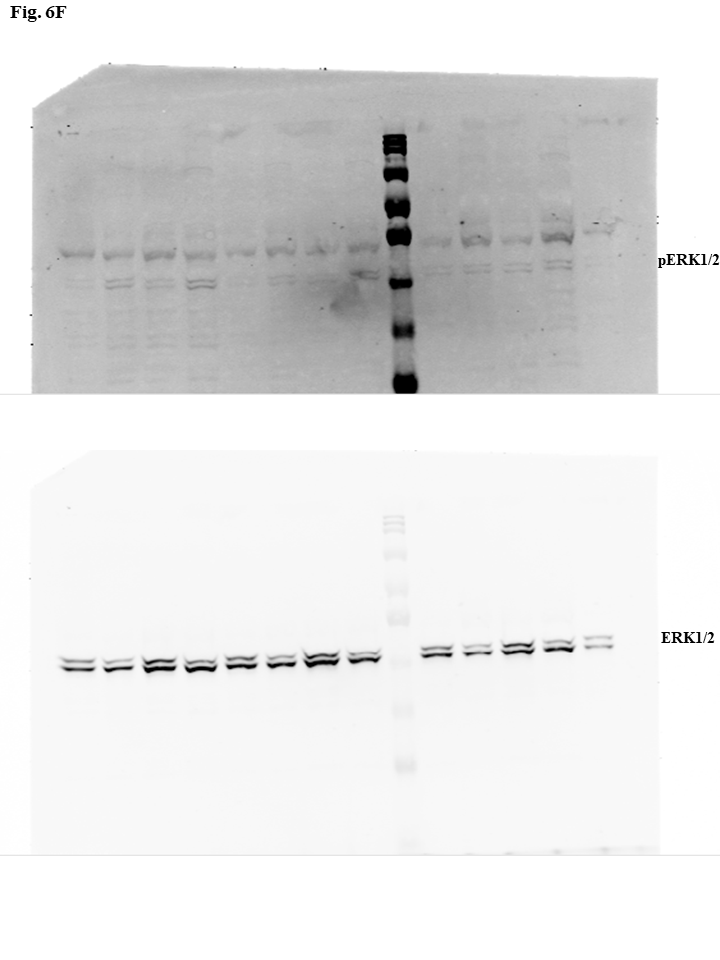


Supplementary fig. 26 Original western blots for the data shown in Fig. 6F. Lanes 1, 2 are shown in Fig. 6F.


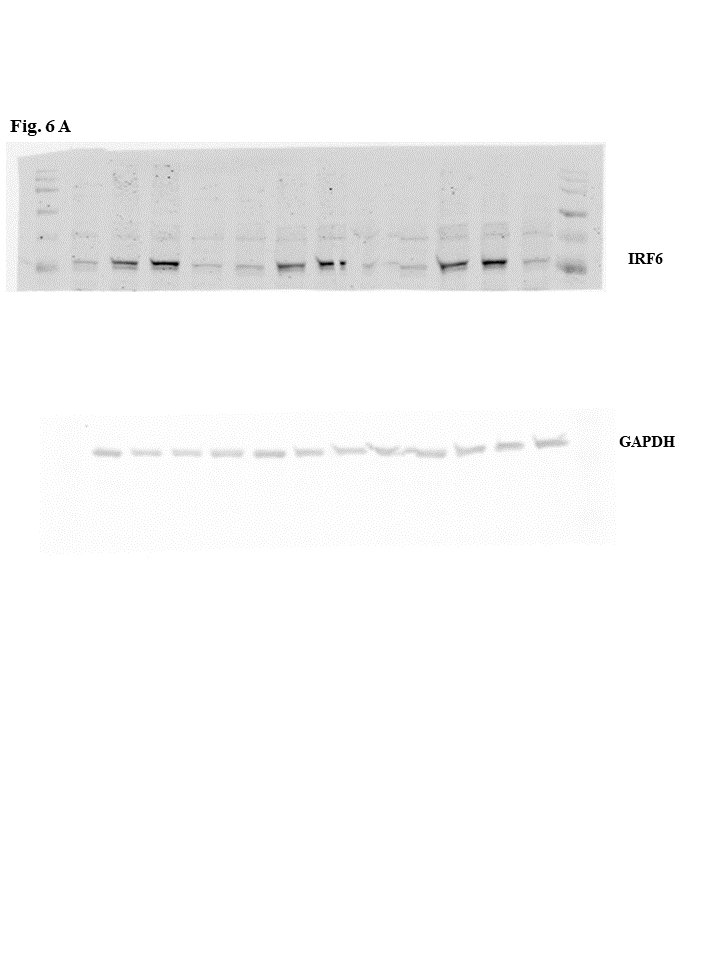


Supplementary fig. 27 Original western blots for the data shown in Fig. 7A. Lanes 5, 6 are shown in Fig. 7A.

.


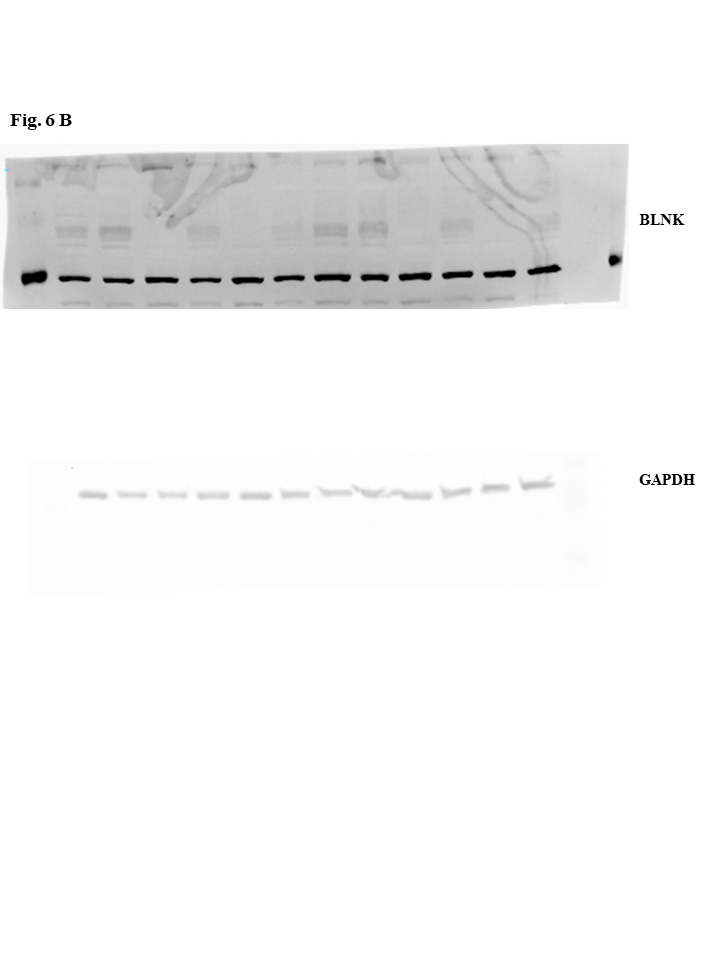


Supplementary fig. 28 Original western blots for the data shown in Fig. 7B. Lanes 9, 10 are shown in Fig. 7B.


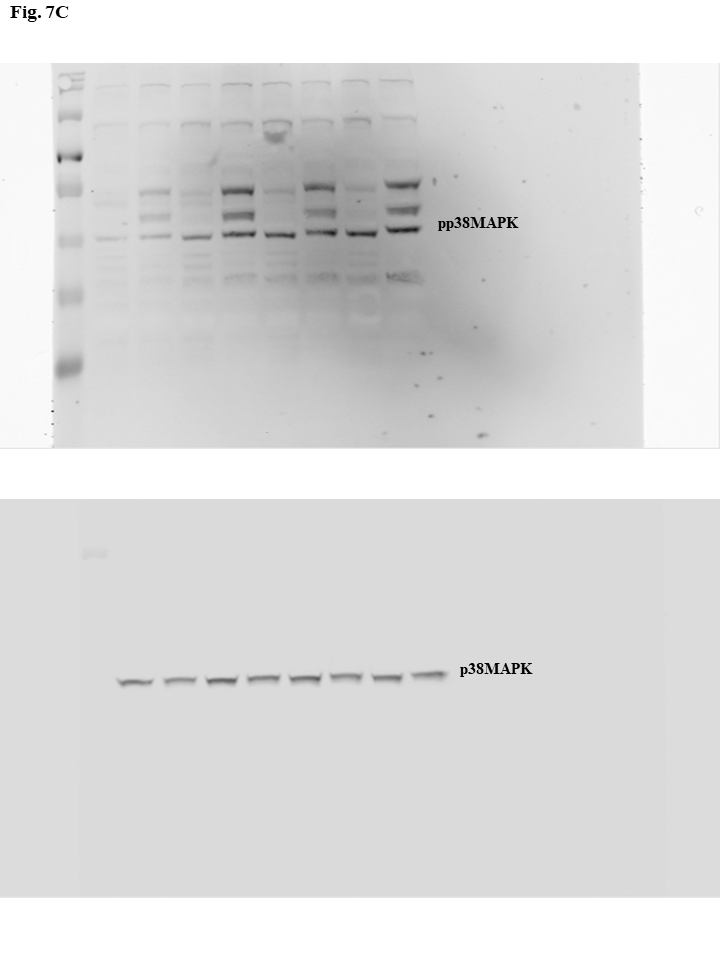


Supplementary fig. 29 Original western blots for the data shown in Fig. 7C. Lanes 1, 2 are shown in Fig. 7C.


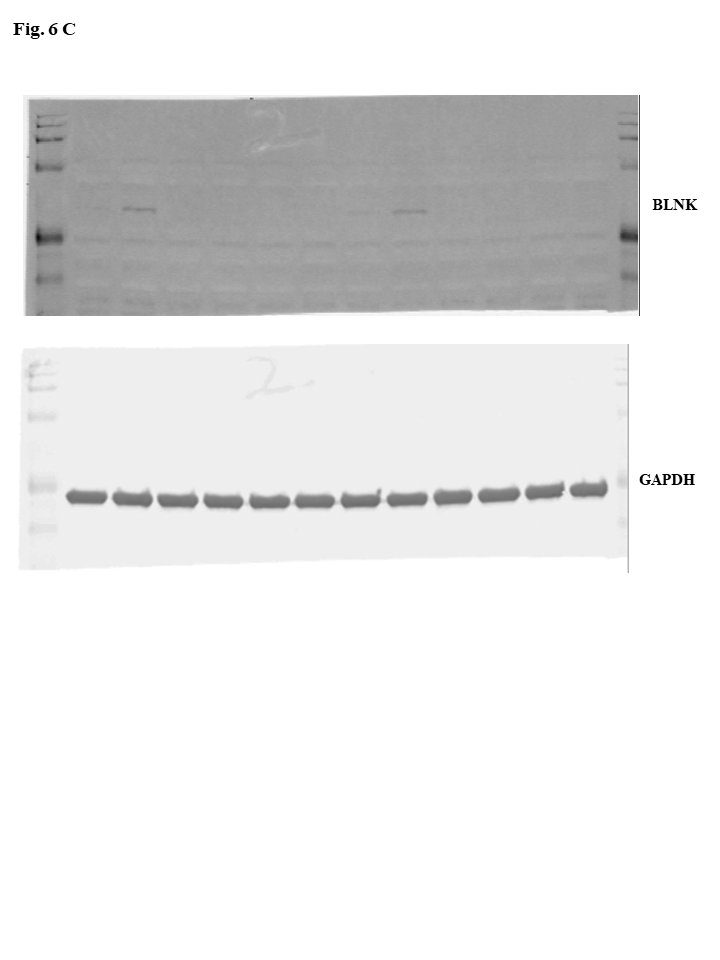


Supplementary fig. 30 Original western blots for the data shown in Fig. 7D. Lanes 1-6 are shown in Fig. 7D.


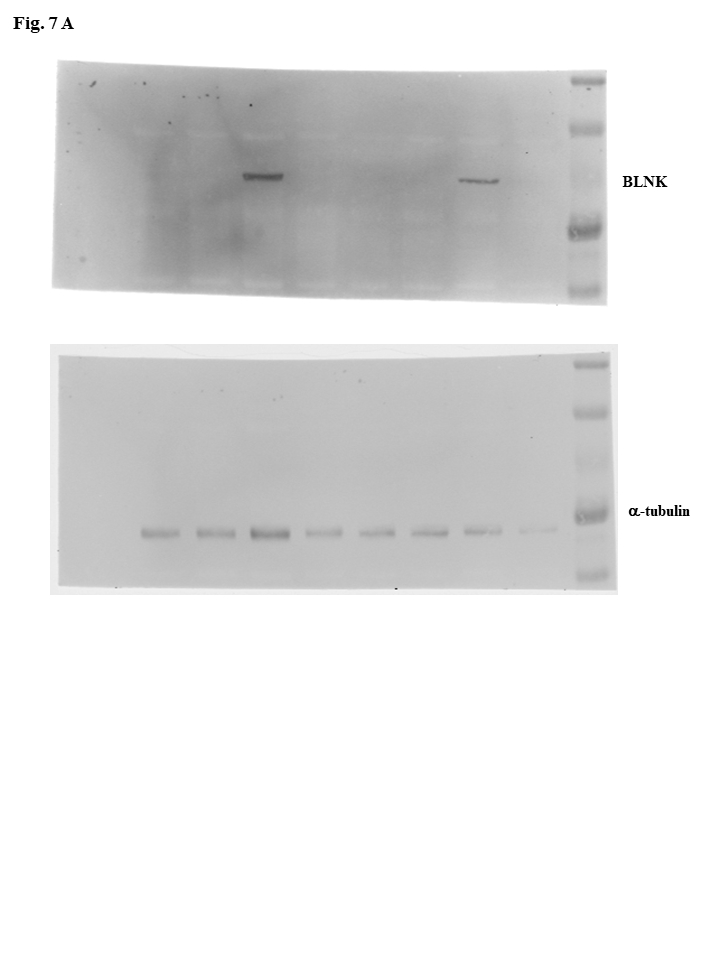
Supplementary fig. 31 Original western blots for the data shown in Fig. 8A. Lanes 4-7 are shown in Fig. 8A. shown in Fig. 8A.
